# Ingested engineered nanomaterials: state of science in nanotoxicity testing and future research needs

**DOI:** 10.1186/s12989-018-0265-1

**Published:** 2018-07-03

**Authors:** Ikjot Singh Sohal, Kevin S. O’Fallon, Peter Gaines, Philip Demokritou, Dhimiter Bello

**Affiliations:** 1 Biomedical Engineering & Biotechnology Program, University of Massachusetts Lowell, Lowell, MA 01854 USA; 2 Natick Soldier Research, Development and Engineering Center, Natick, MA 01760 USA; 3 Department of Biological Sciences, University of Massachusetts Lowell, Lowell, MA 01854 USA; 4 Harvard T.H. Chan School of Public Health, Department of Environmental Health and the Harvard Center for Nanotechnology and Nanotoxicology, Boston, MA 02115 USA; 5 Department of Biomedical and Nutritional Sciences, Zuckerberg College of Health Sciences, University of Massachusetts Lowell, 883 Broadway Street, Dugan 110-S, Lowell, MA 01854 USA

**Keywords:** Ingested nanoparticles, Food grade, Gastrointestinal tract, Caco-2, Titanium dioxide E171, Zinc oxide

## Abstract

**Background:**

Engineered nanomaterials (ENM) are used extensively in food products to fulfill a number of roles, including enhancement of color and texture, for nutritional fortification, enhanced bioavailability, improved barrier properties of packaging, and enhanced food preservation. Safety assessment of ingested engineered nanomaterials (iENM) has gained interest in the nanotoxicology community in recent years. A variety of test systems and approaches have been used for such evaluations, with in vitro monoculture cell models being the most common test systems, owing to their low cost and ease-of-use. The goal of this review is to systematically assess the current state of science in toxicological testing of iENM, with particular emphasis on model test systems, their physiological relevance, methodological strengths and challenges, realistic doses (ranges and rates), and then to identify future research needs and priorities based on these assessments.

**Methods:**

Extensive searches were conducted in Google Scholar, PubMed and Web of Science to identify peer-reviewed literature on safety assessment of iENM over the last decade, using keywords such as “nanoparticle”, “food”, “toxicity”, and combinations thereof. Relevant literature was assessed based on a set of criteria that included the relevance of nanomaterials tested; ENM physicochemical and morphological characterization; dispersion and dosimetry in an in vitro system; dose ranges employed, the rationale and dose realism; dissolution behavior of iENM; endpoints tested, and the main findings of each study. Observations were entered into an excel spreadsheet, transferred to Origin, from where summary statistics were calculated to assess patterns, trends, and research gaps.

**Results:**

A total of 650 peer-reviewed publications were identified from 2007 to 2017, of which 39 were deemed relevant. Only 21% of the studies used food grade nanomaterials for testing; adequate physicochemical and morphological characterization was performed in 53% of the studies. All in vitro studies lacked dosimetry and 60% of them did not provide a rationale for the doses tested and their relevance. Only 12% of the studies attempted to consider the dissolution kinetics of nanomaterials. Moreover, only 1 study attempted to prepare and characterize standardized nanoparticle dispersions.

**Conclusion:**

We identified 5 clusters of factors deemed relevant to nanotoxicology of food-grade iENM: (i) using food-grade nanomaterials for toxicity testing; (ii) performing comprehensive physicochemical and morphological characterization of iENM in the dry state, (iii) establishing standard NP dispersions and their characterization in cell culture medium, (iv) employing realistic dose ranges and standardized in vitro dosimetry models, and (v) investigating dissolution kinetics and biotransformation behavior of iENM in synthetic media representative of the gastrointestinal (GI) tract fluids, including analyses in a fasted state and in the presence of a food matrix. We discussed how these factors, when not considered thoughtfully, could influence the results and generalizability of in vitro and in vivo testing. We conclude with a set of recommendations to guide future iENM toxicity studies and to develop/adopt more relevant in vitro model systems representative of in vivo animal and human iENM exposure scenarios.

**Electronic supplementary material:**

The online version of this article (10.1186/s12989-018-0265-1) contains supplementary material, which is available to authorized users.

## Background

Nanotechnology, a term first used by the late professor Norio Taniguchi in 1974, is the science of manipulating matter at the nanoscale, e.g. atomic, molecular and supramolecular scale [1, 2]. After more than 2 decades of extensive basic nanoscience research, nanotechnology and nano-enabled products have penetrated nearly every field of scientific and economic activity. One such important set of commercial and emerging applications of nanotechnology involves the food industry. Engineered nanomaterials (ENM) in the food industry are used as food additives, in food packaging, as antimicrobials for improving food preservation, for nutrient encapsulation and enhancing bioavailability, as well as in sensing applications for microorganism detection and identification [3–5]. The main function of these ENM additives in the above-mentioned applications is to maintain and/or enhance food texture, flavor, color, consistency, food stability (or preservation), nutrient bioavailability, as well as consumers’ perception of food qualities [6]. We will refer to the ENM used intentionally in foods as ingested ENM, or iENM, to distinguish them from other nanoparticles that may end up in food incidentally, such as those present in airborne pollutants that are deposited on fruits and vegetables, or nanoparticles in food and water that are taken up by or synthesized by plants, or ENM ingested as a result of clearance processes from the lungs following inhalation of airborne ENM.

It is important to note that the term food-grade engineered nanomaterial (referred to as iENM herein) has a particular definition, which we are briefly summarizing here for clarity. According to the European Food Safety authority (EFSA 2011, 2017), the term ENM refers to any intentionally produced material that has one or more dimensions of the order of 100 nm or less or that is composed of discrete functional parts, either internally or at the surface, many of which have one or more dimensions of the order of 100 nm or less, including structures, agglomerates or aggregates, which may have a size above the order of 100 nm but retain properties that are characteristic of the nanoscale [7]. This definition of ENM is consistent with the generally accepted definition of an ENM in nanosciences [8–10]. Within the context of this EFSA guidance document mentioned above, the term “engineered” is equivalent to the term “manufactured” and/or “processed” as used in other reports (e.g. SCENIHR, 2009, 2010 [8, 11]). Only certain (nano) materials are authorized for use in food products and the list varies across countries. Some engineered materials may contain a broad particle size distribution, for which the nanoscale fraction may vary considerably. Engineered materials that contain < 50% nanoparticles by number are not considered nano by certain regulatory agencies (e.g. EFSA), other agencies, such as FDA, do not have such specifications. The 50% cut-point of nanoparticles by number in the definition of an engineered nanomaterial is rather arbitrary and has no toxicological or physiological basis. The term iENM in these cases would strictly refer to only the nanoscale fraction of that material. TiO_2_ E171 is a good example of such a material, as discussed in more detail in later sections.

Ingestion of ENM via dietary intake can be an important pathway of human exposure to nanoparticles. Although iENM are used in various forms in the food industry, those added deliberately to food (as food additives) are likely the primary source of ingested exposure. The dietary consumption of iENM in developed countries is not known with accuracy, but it is estimated to be considerable. An early study estimated an ingestion uptake of ~ 10^12^ particles/person per day, which consists mainly of titanium dioxide (TiO_2_), colloidal silica, and mixed silicates [12]. A more recent survey of TiO_2_ consumption patterns from food, dietary supplements and toothpaste in the Dutch population estimated mean long-term intake of TiO_2_ ranges from 0.06 mg/kg body weight (bw)/day in the elderly (≥70-years-old) to 0.67 mg/kg bw/day in children (2–6-year-old) [13]. Yang et al. [14] reported the occurrence of food-grade silicon dioxide nanoparticles in foods and orally-consumed goods such as taco seasoning, vitamin tablets, cappuccino, and toothpaste, at levels of 1.3–16.2 mg Si/g product. Dunkin’ Donuts USA, Inc., which had been using titanium dioxide as part of the powdered sugar coating on donuts, agreed to remove the potentially harmful nanomaterial in 2015 due to pressure from shareholders after an independent study from the San-Francisco-based As You Sow confirmed their presence [15]. Chen et al. [16] found that greater than 96% of TiO_2_ can be found in the sugar coating of chewing gums and only 0.15–0.38% can be attributed to the gum base [17]. Moreover, the authors further estimated that chewing a sugar-coated gum for 10 min could lead to an intake of as much as 5.1 mg of TiO_2_ particles. Significant variation in diets, food preferences, and the content of such iENM additives in different food products produces a wide range of estimated daily human consumption of TiO_2_, silicon dioxide (SiO_2_), or any other iENM for that matter. In extreme cases, the use of specific products, such as salad dressing containing TiO_2_ as a whitening agent, can lead to more than a 40 fold increase in the daily average intake [17]. It should be mentioned that food-grade TiO_2_ (E171) used in these products as a whitening agent contains less than 30% nanoscale particles, with the remainder being in the 150–400 nm (nm) range [18]. Such widespread occurrence of iENM in common food products necessitates studying their impact on the GI tract and human health in general.

The interest in evaluating nanotoxicity of iENM has been recently renewed, as reflected in the number of publications on this topic (detailed in the Results and Discussion section). Various test models have been used, of which cell monocultures and certain co-cultures predominate. In vitro assays provide quick and inexpensive approaches to testing toxicity of an ENM, including iENM, in a specific cell culture system. However, interpretation of in vitro results and their relevance to in vivo risk assessment is less straightforward [19, 20]. To date, little is known about the toxicokinetic and toxicodynamic processes, as well as in situ biotransformation kinetics following oral exposure, particularly in relation to ingestion of iENM that are present in food. Several in vivo studies have been conducted in rats to determine the biodistribution, elimination and toxicity of iENM [21–25]. Recently, however, it has been suggested that the impact of iENM on the GI tract should be re-evaluated because of significant differences in the physiology and nutrient uptake of the GI tract between humans and rats [26–29].

The main objective of this review is to summarize and critically evaluate the nanotoxicology literature on iENM, especially direct iENM food additives, in the context of test systems, methodological approaches, persistent challenges, and critical research gaps that require addressing in the future. Although the findings of these studies have been summarized, the focus of our review is to critically assess how the findings may have been impacted by the selection of test materials, methodological issues such as dispersion and dosimetry, the dose ranges employed, and iENM dissolution behavior. We conclude with recommendations for improving the design of future studies.

## Methods

**Fig. 1:**
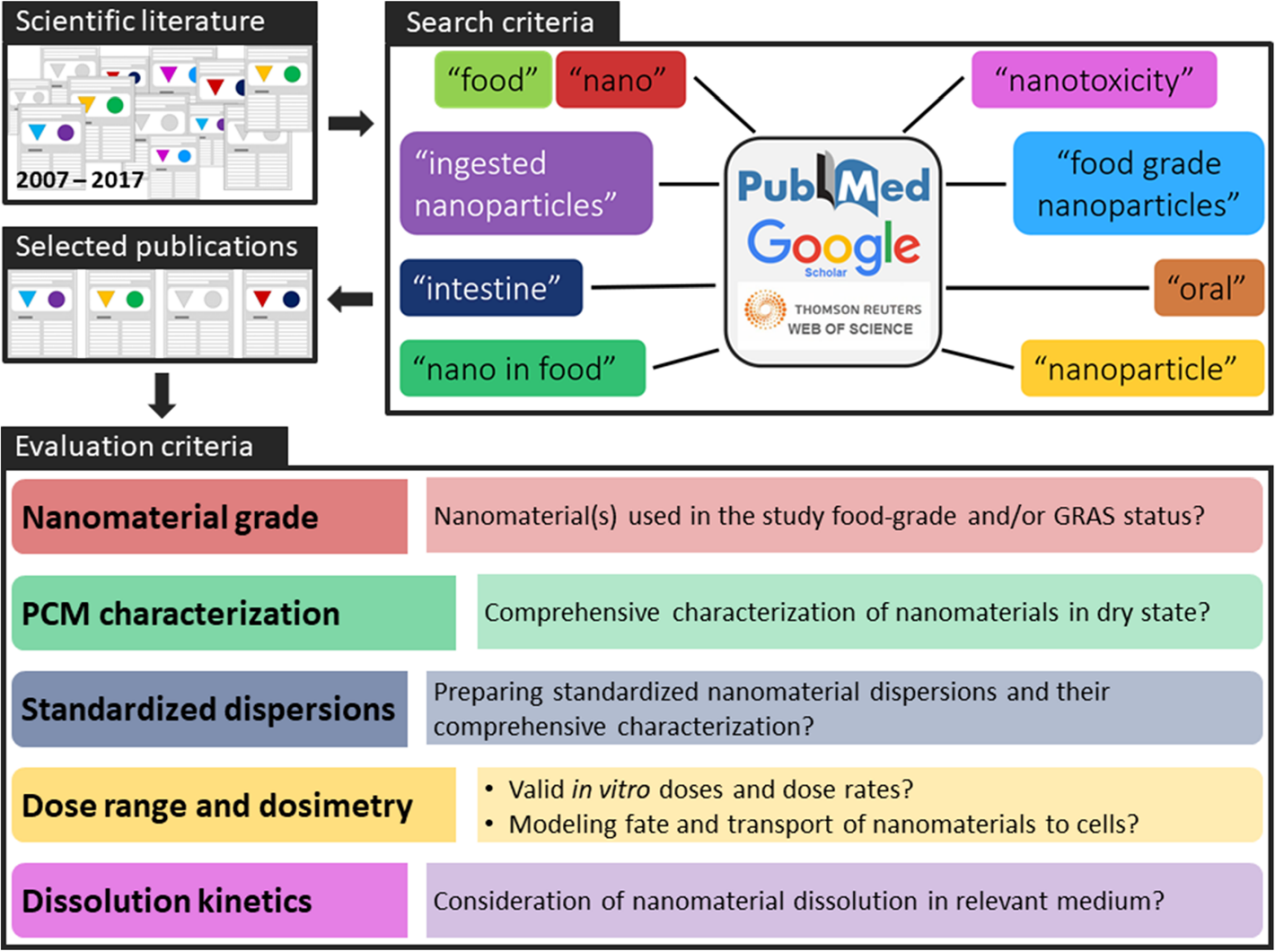
Publication searching schematic. Approximately 650 publications from 2007 to 2017 were screened using relevant terms to identify in vitro and in vivo publications relevant to ingested nanotoxicity theme. The selected publications were evaluated on 5 domains, the details of which are discussed in the “Methods” section

A combination of the terms “nano”, “nanoparticle”, “food”, “food grade nanoparticles”, “nano in food”, “toxicity”, “nanotoxicity”, “oral”, “intestine”, and “ingested nanoparticles” were used in Google Scholar, PubMed, and Web of Science search engines to identify scientific publications on toxicity of iENM (Fig. 1). The search process focused on toxicity studies using nano/materials such as titanium dioxide, silicon dioxide, iron oxide and zinc oxide that were directly added to food products. Furthermore, we also used Google search engine to search for additional magazine articles related to identification of iENM in food products. Additional publications were traced from the references section of other articles and from our own collection of nano literature dating back to 2007. Only peer-reviewed scientific publications that were published in “English” were included in the analysis. A total of 650 peer-reviewed articles were identified, of which 39 met the inclusion criteria. Each scientific publication was thoroughly reviewed, and information was extracted on 5 domains related to aspects of the methodology, as detailed in Fig. 1. Other published reviews have argued for the need to intensify research on the toxicology and risk assessment of iENM [30]. For publications that presented both in vitro and in vivo data, each component was evaluated separately.

### Using food-grade nanomaterials

Each publication was evaluated based on whether the nanomaterials used in the study were certified as food-grade (i.e. allowed to be used as food additives), or not. Publications that did not specify nanomaterial type, or for which we were unable to collect that information based on the data reported in the manuscript, were denoted as lacking that information (“not reported”).

### Comprehensive physicochemical and morphological (PCM) characterization of ENM in dry state

Each publication was evaluated for whether the studied nanomaterials’ physicochemical and morphological properties were comprehensively characterized in the dry state or not. Any reference to previous characterization data for the same nanomaterials was considered as satisfactory. We followed the European Food Safety Authority (EFSA) guidelines to define the term “comprehensive”, which states adequate characterization of engineered nanomaterials used in food products should include chemical composition, particle size/size distribution, physical form and morphology, particle and mass concentration, specific surface area, surface chemistry, surface charge, redox potential, and chemical reactivity/catalytic activity [7].

### Standardized nanoparticle dispersions and their characterization

Each publication was evaluated for whether the study used standardized protocols to disperse test nanomaterials and characterize the dispersions. A “standard dispersion protocol” was defined as a protocol that follows best available science on nanoparticle dispersion, characterization, and fate and transport modeling [31–33]. Such protocols describe in detail information such as the make of the sonicator plus amplitude and power used, sample volume, dispersing media, measured energy density, and energy delivered to prepare stable nanoparticle dispersions, so that the protocol could be reproduced across labs. Characterization of nanoparticle dispersions was considered minimally sufficient when size and charge distribution analysis data, obtained by DLS (dynamic light scattering) or NTA (Nanoparticle tracking analysis) and electron microscopy, were provided together with the polydispersity index (PdI). In addition, effective density of agglomerates, an experimentally measured parameter critical for in vitro dosimetry modeling, is another important property of nanoparticle dispersions that should be measured experimentally and reported [33–35].

### Dosimetry considerations: Dose range, rate, and the rationale

Each publication was evaluated for the dose ranges used and whether such ranges were justified or accounted for based on human daily dietary intake data for the respective nanomaterials. In addition, in vitro studies were evaluated for dosimetry considerations, particularly whether fate and transport models were used to calculate the delivered dose to cells as a function of time. Dose rate (amount of dose per unit of time) was also considered and assessed in each of those studies.

### Dissolution kinetics

Each publication was evaluated for considerations of dissolution kinetics or biokinetics (for in vivo studies) of test nanomaterials. For in vitro studies, the evaluation was based on data provided regarding ionic release of test nanomaterials’ over time in relevant test media. For in vivo studies, the evaluation was based on data provided on ENM ion release over time in simulated or actual digestive fluids and/or accumulation of ions in circulation.

## Results and discussion

### Food-grade engineered nanomaterials in various food products

Nanomaterials are available in a variety of grades for their intended use in different products or applications. For example, pharmaceutical grade is a standard of purity that has been established by any recognized pharmacopeia, including US Pharmacopeia (USP), National Formulary (NF), British Pharmacopeia, or European Pharmacopeia (EP), which is suitable for use as an active or inactive drug, biologic, or reagent. Generally, the products are required to be 97–101% pure depending on their application and must contain ≤0.1% of bacteria. Engineered nanomaterials and their bulk counterparts used in or in-contact with food are considered direct or indirect food additives, respectively. In the United States, iENM are required to meet the food-grade guidelines issued by the Food and Drug administration (FDA), which are typically less rigorous than the specifications for pharmaceutical grade. Such iENM are mostly GRAS (Generally Recognized as Safe) materials. GRAS status is assigned by the FDA to a product that is not known to be hazardous to health and thus approved for use in foods. From our searches and interactions with iENM vendors, it appeared that companies are permitted to “self-affirm” GRAS status for certain iENM products. Furthermore, there appears to be a notable lack of rigorous oversight on the granting of ‘GRAS’ status for iENM by the FDA.

**Fig. 2:**
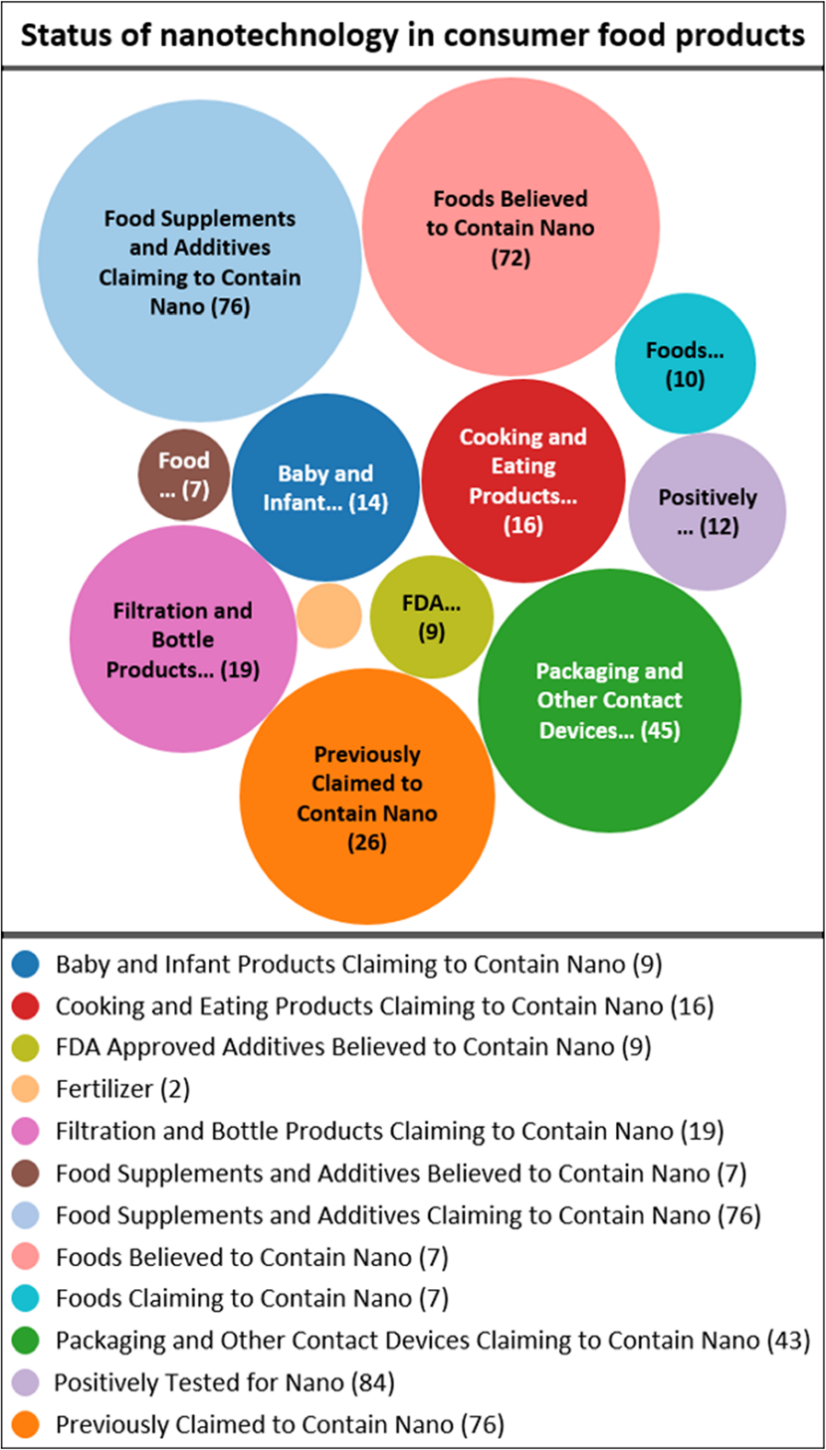
Bubble map of status of nanotechnology in various consumer food products registered in the database as of December 17th, 2017 (adapted and reprinted with permission from Center for Food Safety)

Engineered nanoparticles are used widely in a variety of products. In the USA, the Center for Food Safety maintains a comprehensive database [36] of consumer products in the food industry believed to contain nanoparticles (NP). The database focusses exclusively on food and food contact products, covering over 300 products and 40 different types of nanomaterials. The database was evaluated as part of this review and the results have been summarized in Fig. 2. Over 70 food products contained iENM based on independent testing and/or labels. In addition, iENM were present in 14 baby and infant products and 16 cooking products [36]. A sizeable category of 45 products contained iENM intended for packaging. It is also apparent from Fig. 2 that independent and more rigorous testing of such products to confirm the presence of iENM, along with their concentration, chemical composition, and size distributions of primary particles vs. agglomerates is needed. The most common iENM used directly in food products are TiO_2_ (only part of the product is in the nanoscale, as noted above), SiO_2_ (silicon dioxide), iron oxides (Fe_2_O_3_), zinc oxide (ZnO) and silver (Ag). Table 1 summarizes the types of foods containing each type of iENM, with product examples and reported mass concentrations for each product. These materials are or contain at least one product that fulfills the strict definition of an engineered nanomaterial with regards to primary particle size and synthesis/processing method. TiO_2_ E171 for example has only ~ 1/3 of particles in the nanoscale. However, our own analysis of other TiO_2_ used in foods (and certified as food-grade E171 equivalent) revealed that some of them may contain > 50% nanoscale particles and would therefore meet the strict definition of an iENM. Notable in such analysis is the lack of information on primary particle size distribution and agglomerates, which is useful for in vitro iENM testing relevant to the nano fraction. It is also interesting to note how each iENM is used in the different products. For example, TiO_2_ is used frequently as a whitening agent, SiO_2_ (silica made by wet processes or flame pyrolysis and not to be confused with crystalline silica) is used as filler, and both ZnO and Fe_2_O_3_ are used as food supplements. Silver has been used as a direct food additive in various colloidal silver drinks, whereas other Ag uses are in food contact applications. This analysis also reveals the prominence of nano ingredients such as TiO_2_ and SiO_2_ in several foods frequently consumed by children.

**Table 1:** Most frequent ingested engineered materials and nanomaterials (iENM) used in foods together with products and their concentrations, as reported in the literature

Nanomaterial	Commercial products	Concentration	References
Titanium dioxide	Dickinson’s Coconut Curd Hostess Powdered Donut Kool Aid Blue Raspberry M&Ms. Chocolate Candy	3.59 μg Ti/mg 2.42 μg Ti/mg 1.69 μg Ti/mg 1.25 μg Ti/mg	[144]
Silicon dioxide	Multivitamins Instant noodles tandoori Taco seasoning mix	6.4 μg Si/mg 6.0 μg Si/mg 5.3 μg Si/mg	[145]
Zinc oxide	Breakfast cereals Nutrition drinks Nutrition bars	Not available	[146]
Iron oxides	Capsules Fish and crustacean paste Sauces (excluding tomato-based sauces)	Not available	[147]
Silver	Sovereign Silver MesoSilver Nanoceuticals Silver 22 Advanced Colloidal Silver	10 μg Ag/mL 20 μg Ag/mL 22 μg Ag/mL 20 μg Ag/mL	Natural Immunogenics Corp. Purest Colloids, Inc. RBC Life Sciences®, Inc. Utopia Silver Supplements

Specifications regulating use of each iENM as a food additive also are unique. Titanium dioxide (TiO_2_) is allowed as a color additive (whitening agent) in food by the FDA, provided the added product conforms to the recommended specifications as described in Title 21 [37] of the e-CFR (electronic Code of Federal Regulations) and does not exceed 1% by weight of the food. The FDA has no specific guidelines for use of TiO_2_ as a dietary supplement or as an antimicrobial agent in food products. Silicon dioxide is permitted by FDA as a “food additive for direct addition to food for human consumption” provided that it is manufactured by vapor phase hydrolysis [38], does not exceed 2% by weight of the food, and conforms to the recommended specifications. Silica’s intended use as an anticaking agent is subject to the following conditions: (i) it is only permitted in foods in which the additive has been demonstrated to have an anticaking effect, (ii) it can be used in an amount not more than what is reasonably required to produce its intended effect, and (iii) it can be used in an amount not to exceed 2% by weight of the food. Iron oxide and its hydrated forms are allowed by the FDA as a color additive in food, provided it conforms to the recommended specifications [39] and does not exceed 0.1% by weight of the final food product. In the USA, ZnO is also allowed as a color additive in foods, as well as in cosmetics, provided it is manufactured by the French process (described as the indirect process whereby zinc metal isolated from the zinc-containing ore is vaporized and then oxidized), conforms to the recommended specifications [39], and is used in accordance with good manufacturing practices. Of note, these regulations are for their microscopic bulk materials and not specifically for the nanomaterials themselves.

#### Model test systems

Sixteen of the 24 in vitro studies reviewed utilized Caco-2 monocultures or a sub-clone of Caco-2 known as C2BBe1 cells, Table 2), both of which, even though originating from human epithelial colorectal adenocarcinoma cells, can be induced to differentiate into morphologically and functionally mature cells that resemble the enterocytes lining the small intestine. Although it has been suggested that C2BBe1 monolayers are more representative of the small intestinal epithelium than Caco-2 due to similar transepithelial electrical resistance, morphological homogeneity and BB myosin I expression levels similar to that of a human enterocyte, only 2 studies were found using them [40, 41]. Additionally, the epithelial cell line used in vitro should be allowed to grow, form tight junctions and differentiate to enterocytes to form an intact barrier – representative of the GI tract epithelium, which is verified by measuring TEER (Trans-epithelial electrical resistance) values and expression of tight junction proteins before nanotoxicological assessment [42–44]. Other in vitro models include the cell lines representative of gastric epithelium (GES-1), mucus-secreting cells (HT29-MTX), colon epithelium (SW480, DLD-1), and mucus-secreting colon epithelium (NCM460). Of note was the use of MET-1 bacterial community to represent an in vitro model of gut microbial community [45]; and combinations of Caco-2/HT29-MTX or Caco-2/Raji-B cell lines in co-culture models to represent mucus-secreting epithelium and follicle-associated epithelium, respectively [46].

**Table 2:** Overview of the key findings regarding the state of science in in vitro nanotoxicity testing of food-grade nanomaterials, categorized by nanomaterial type

First author	Year	Test system	Dose range (administered)	Nanomaterial grade	PCM characterization	Standardized dispersion and characterization	Dose range rationale and dosimetry	Dissolution biokinetics	Main conclusions from study	Ref
Titanium dioxide										
Kirsten Gerloff	2009	Caco-2 cells	20 and 80 μg/cm^2^ for 4 h and 24 h	Not reported	Manufacturer provided	No standard dispersion protocol specified	Not reported	Not reported	Food-related nanoparticles potentially hazardous. All nanoparticles (TiO_2_, SiO_2_, CB, and ZnO) except MgO exhibit cytotoxicity. ZnO and SiO_2_ induce DNA damage while SiO_2_ and CB cause glutathione depletion	[80]
Brian A. Koeneman	2010	Caco-2 cells	Acute dose of 10, 100 and 1000 μg/ml. Chronic dose of 100 and 1000 μg/ml	Not reported	Primary particle size and SSA (provided by manufacturer), SEM, DLS, zeta potential	Not reported	Not reported	Not reported	TiO_2_ nanoparticles can potentially translocate through epithelial lining (at low levels) by transcytosis and induce sub-lethal effects – microvilli reorganization and intracellular calcium increase in Caco-2 cells	[56]
Kirsten Gerloff	2012	Caco-2 cells	20 and 80 μg/cm^2^ for 4 h and 24 h	Not reported	XRD, SSA by BET method, XRF analysis, TEM, and DLS in DI water and cell culture media	No standard dispersion protocol specified	Not reported	Not reported	Anatase/rutile TiO_2_ nanoparticles show higher toxicity per unit surface area than pure anatase	[68]
Matthieu Fisichella	2012	Caco-2 cells	10 to 100 μg/ml for 4 h, 24 h and 72 h	TiO_2_ STNPs widely used in sunscreens	DLS in DI water and culture medium, zeta potential, TEM	Not reported	Dose range based on accidental high exposures, but dosimetry not taken into consideration	Not reported	Surface-treated TiO_2_, which have a strong tendency to agglomerate in complex media, show no toxic effects on Caco-2 cells after exposures up to 72 h	[74]
Yun Zhao	2013	Human primary epidermal keratinocytes	50 fg/ml to 500 μg/ml for 24 h	Not reported	TEM, DLS	No standard dispersion protocol specified	Not reported	Not reported	TiO_2_ nanoparticles induce autophagy in addition to cell viability loss in human primary epidermal keratinocytes	[58]
Christie McCracken	2013	C2BBe1 cells	10 μg/cm^2^ for short-term (24 h) and long-term (29 exposure cycles) exposure	Not reported	DLS, zeta potential, TEM, DRIFTS, XRD	No standard dispersion protocol specified	Not reported	AAS to measure Zn^2+^ from ZnO nanoparticles in stomach phase	C2BBe1 cells internalize TiO_2_, SiO_2_ and ZnO nanoparticles but show mild toxicity only upon exposure to ZnO nanoparticles. TiO_2_ nanoparticles exposed to simulated digestion environment induce mild toxic effects	[59]
Isabella De Angelis	2013	Caco-2 cells	1, 2.5, 5, 10 and 20 μg/cm^2^ for 6 h and 24 h	Not reported	DLS, zeta potential, SEM, TEM, ICP-MS	No standard dispersion protocol specified	Not reported	ICP-MS to measure amount of Zn or Ti in cells	ZnO nanoparticles, in contrast to TiO_2_ nanoparticles, induce significant toxicity in Caco-2 cells by increasing intracellular ROS levels, pro-inflammatory cytokine (IL-8) and releasing Zn^2+^ ions	[60]
Kirsten Gerloff	2013	Caco-2 cells	0.3125, 1.25, 5, 20 and 80 μg/cm^2^ for 4 h and 24 h	Not reported	TEM, ICP-OES, DLS, SLD	No standard dispersion protocol specified	Not reported	Not reported	Undifferentiated Caco-2 cells more sensitive to the toxic effects exerted by SiO_2_ and ZnO nanoparticles than differentiated Caco-2 cells	[127]
Xin-Xin Chen	2013	Caco-2 cells, GES-1 cells	10, 25, 50, 100 and 200 μg/ml for 24 h	Nanoparticles extracted from commercially available chewing gums	XRD, TEM-EDS, SEM, NTA	Not reported	Not reported	Not reported	More than 93% of TiO_2_ in chewing gums is in nano form and ~ 95% of nano-TiO_2_ particles end up being swallowed. Nano-TiO_2_ relatively safe for GES-1 and Caco-2 cells	[16]
Zhangjian Chen	2014	V79 cells	5, 10, 20, 50 and 100 μg/ml for 6 h, 24 h and 48 h	Not reported	Previously characterized [96]	No standard dispersion protocol specified	Not reported	Not reported	TiO_2_ nanoparticles induce significant increase in DNA strand breaks, % Tail DNA and HPRT gene locus mutation frequency	[62]
James J. Faust	2014	C2BBe1 cells	0.35, 3.5 and 35 μg/ml for 24 h	Food grade TiO2 and TiO2 extracted from chewing gums	XPS, XRD, TEM, DLS, zeta potential	No standard dispersion protocol specified	Not reported	Not reported	Food grade TiO2 nanoparticles disrupt brush border epithelium independent of sedimentation	[26]
Emilie Brun	2014	Caco-2 cells, co-culture of Caco-2 and HT29-MTX cells (mucus-secreting epithelium), co-culture of Caco-2 and Raji B cells (follicle-associated epithelium)	50 and 2000 μg/ml for 48 h	Not reported (self-synthesized)	SSA by BET, XRD, TEM, agglomeration state (DLS), zeta potential, XAS	Nanoparticle suspensions pulse sonicated at 28% amplitude – corresponding power measured using a calorimetric procedure [104]	Dose range based on worst case scenario, but dosimetry not taken into consideration	Not reported	TiO_2_ nanoparticles pass through follicle-associated epithelium model only and their intracellular accumulation depends highly on the cell model – higher in Goblet and M cells than in enterocytes. Intracellular TiO_2_ does not dissolve and shows higher biopersistance	[46]
Constantinos Gitrowski	2014	Caco-2 cells	1 mg/L for 0 h, 2 h, 4 h, 6 h, 8 h and 24 h	Not reported	TEM and NTA in water	No standard dispersion protocol specified	Not reported	Not reported	Caco-2 cells show characteristic active uptake of Ti from TiO_2_ nanoparticle exposures, which is dependent on the crystal form of the nanomaterial	[109]
Birgit J. Teubl	2015	Buccal mucosa (ex vivo), Human buccal epithelial cells (TR146)	50, 100, 150 and 200 μg/ml for 4 h and 24 h	One pigment-grade TiO_2_. Not reported for the other two TiO_2_	TEM, DLS, FTIR, laser diffraction analysis, surface hydrophobicity by RB adsorption method	Nanoparticle suspensions ultra-sonicated from 1 to 24 h to evaluate the optimal method to ensure lowest mean particle sizes	Not reported	Not reported	TiO_2_ nanoparticles tend to aggregate in saliva but available nano-TiO_2_ gets internalized in the oral cavity within 10 min. Although no effect on viability and membrane integrity, internalized TiO_2_ triggers ROS production in the cells of buccal epithelium after short-time incubation	[148]
Magdiel I. Setyawati	2015	SW480, DLD-1 and NCM460 cells	62.5, 250 and 1000 μM for 24 h	Not reported	FETEM, hydrodynamic size (DLS), zeta potential	No standard dispersion protocol specified	Not reported	Not reported	Among ZnO, TiO_2_ and SiO_2_, ZnO nanoparticles were the most cytotoxic to all three intestinal cell types. Different cellular responses among the three cell types owes to their different genetic landscape	[64]
Zheng-Mei Song	2015	Caco-2 cells	50 and 200 μg/ml for 24 h	Food additive TiO_2_ and regular TiO_2_	XRF, XRD, TEM, hydrodynamic size (DLS), zeta potential, FTIR spectroscopy	Not reported	Not reported	Not reported	Native and digestion fluid pretreated TiO_2_ nanoparticles get internalized by Caco-2 cells but not toxic to Caco-2 cells/monolayers. The possibility of TiO_2_ nanoparticles translocating through Caco-2 monolayers is low	[99]
Saeko Tada-Oikawa	2016	THP-1 and Caco-2 cells	1, 10, 25 and 50 μg/ml for 24 h and 72 h	Not reported	Hydrodynamic size (DLS), TEM, zeta potential	Nanoparticle suspensions were sonicated based on a standardized protocol [101]	Not reported	Not reported	Anatase TiO_2_ nanoparticles induce inflammatory response by upregulating IL-1β and IL-8 production in THP-1 and Caco-2 cells, respectively	[69]
Maria G. Ammendolia	2017	HT29 cells	1, 2.5, 5 and 20 μg/cm^2^ for 6 h, 24 h and 48 h	Not reported	TEM, SEM, hydrodynamic diameter (DLS), PdI. SSA and purity (provided by manufacturer)	No standard dispersion protocol specified	Not reported	Not reported	TiO_2_ nanoparticles do not induce cytotoxicity or changes in mitochondrial membrane potential but cause dose-dependent oxidative stress that decreases at 24 h. TiO_2_ nanoparticles, in combination with IGF-1, induce higher cell proliferation as compared to TiO_2_ nanoparticles alone	[125]
William Dudefoi	2017	MET-1 bacterial community	100 and 250 ppm for 48 h	Two food-grade TiO_2_ and one P25 TiO_2_	TEM, XRD, isoelectric point, SSA by BET, XPS	Not applicable	Dose range based on the amount of TiO_2_ found in the intestine after ingestion of 1–2 pieces of gum or candy	Not applicable	TiO_2_ nanoparticles do not significantly alter the human gut microbiota by showing little impact on a defined anaerobic gut microbial community MET-1, as assessed through bacterial respiration, fatty acid profiles and phylogenetic composition	[45]
Silicon dioxide										
Kirsten Gerloff	2009	Caco-2 cells	20 and 80 μg/cm^2^ for 4 h and 24 h	Not reported	Manufacturer provided	No standard dispersion protocol specified	Not reported	Not reported	Food-related nanoparticles potentially hazardous. All nanoparticles (TiO_2_, SiO_2_, CB, and ZnO) except MgO exhibit cytotoxicity. ZnO and SiO_2_ induce DNA damage while SiO_2_ and CB cause glutathione depletion	[80]
Helge Gehrke	2012	HT29 cells	0.03, 0.31, 1.56, 3.13, 15.6, 31.3, 93.8 and 156.3 μg/cm^2^ for 24 h, 48 h and 72 h	Not reported	TEM, DLS, zeta potential	No standard dispersion protocol specified	Not reported	Not reported	SiO_2_ nanoparticle stimulate HT29 cell proliferation whereas cytotoxicity depends on its concentration and size, and FCS (Fetal calf serum) content of the cell culture medium	[57]
Christie McCracken	2013	C2BBe1 cells	10 μg/cm^2^ for short-term (24 h) and long-term (29 exposure cycles) exposure	Not reported	DLS, zeta potential, TEM, DRIFTS, XRD	No standard dispersion protocol specified	Not reported	AAS to measure Zn^2+^ from ZnO nanoparticles in stomach phase	C2BBe1 cells internalize TiO_2_, SiO_2_ and ZnO nanoparticles but show mild toxicity only upon exposure to ZnO nanoparticles. TiO_2_ nanoparticles exposed to simulated digestion environment induce mild toxic effects	[59]
Kirsten Gerloff	2013	Caco-2 cells	0.3125, 1.25, 5, 20 and 80 μg/cm^2^ for 4 h and 24 h	Not reported	TEM, ICP-OES, DLS, SLD	No standard dispersion protocol specified	Not reported	Not reported	Undifferentiated Caco-2 cells more sensitive to the toxic effects exerted by SiO_2_ and ZnO nanoparticles than differentiated Caco-2 cells	[127]
Yi-Xin Yang	2014	GES-1 cells, Caco-2 cells	10, 25, 50, 100, 200, 400 and 600 μg/ml for 24 h, 48 h and 72 h	Food additive SiO_2_ nanoparticles	XRD, TEM, SSA by BET, hydrodynamic size (DLS), zeta potential, XRF, FTIR	No standard dispersion protocol specified	Not reported	Not reported	At higher concentrations, food additive SiO_2_ nanoparticles enter cells and inhibit cell growth by cell cycle arrest	[128]
Magdiel I. Setyawati	2015	SW480, DLD-1 and NCM460 cells	62.5, 250 and 1000 μM for 24 h	Not reported	FETEM, hydrodynamic size (DLS), zeta potential	No standard dispersion protocol specified	Not reported	Not reported	Among ZnO, TiO_2_ and SiO_2_, ZnO nanoparticles were the most cytotoxic to all three intestinal cell types. Different cellular responses among the three cell types owes to their different genetic landscape	[64]
Zinc oxide										
Kirsten Gerloff	2009	Caco-2 cells	20 and 80 μg/cm^2^ for 4 h and 24 h	Not reported	Manufacturer provided	No standard dispersion protocol specified	Not reported	Not reported	Food-related nanoparticles potentially hazardous. All nanoparticles (TiO_2_, SiO_2_, CB, and ZnO) except MgO exhibit cytotoxicity. ZnO and SiO_2_ induce DNA damage while SiO_2_ and CB cause glutathione depletion	[80]
Christie McCracken	2013	C2BBe1 cells	10 μg/cm^2^ for short-term (24 h) and long-term (29 exposure cycles) exposure	Not reported	DLS, zeta potential, TEM, DRIFTS, XRD	No standard dispersion protocol specified	Not reported	AAS to measure Zn^2+^ from ZnO nanoparticles in stomach phase	C2BBe1 cells internalize TiO_2_, SiO_2_ and ZnO nanoparticles but show mild toxicity only upon exposure to ZnO nanoparticles. TiO_2_ nanoparticles exposed to simulated digestion environment induce mild toxic effects	[59]
Isabella De Angelis	2013	Caco-2 cells	1, 2.5, 5, 10 and 20 μg/cm^2^ for 6 h and 24 h	Not reported	DLS, zeta potential, SEM, TEM, ICP-MS	No standard dispersion protocol specified	Not reported	ICP-MS to measure amount of Zn or Ti in cells	ZnO nanoparticles, in contrast to TiO_2_ nanoparticles, induce significant toxicity in Caco-2 cells by increasing intracellular ROS levels, pro-inflammatory cytokine (IL-8) and releasing Zn^2+^ ions	[60]
Kirsten Gerloff	2013	Caco-2 cells	0.3125, 1.25, 5, 20 and 80 μg/cm^2^ for 4 h and 24 h	Not reported	TEM, ICP-OES, DLS, SLD	No standard dispersion protocol specified	Not reported	Not reported	Undifferentiated Caco-2 cells more sensitive to the toxic effects exerted by SiO_2_ and ZnO nanoparticles than differentiated Caco-2 cells	[127]
Yanli Wang	2014	GES-1 cells, Neural stem cells	15 μg/ml for 24 h	Not reported	XRD, TEM, XRF, hydrodynamic size (DLS) in water and cell culture medium, zeta potential	Not reported	Not reported	Not reported	Higher rate of dissolution of ZnO nanoparticles in the presence of Vitamin C aggravate the toxic effects of ZnO nanoparticles	[63]
Magdiel I. Setyawati	2015	SW480, DLD-1 and NCM460 cells	62.5, 250 and 1000 μM for 24 h	Not reported	FETEM, hydrodynamic size (DLS), zeta potential	No standard dispersion protocol specified	Not reported	Not reported	Among ZnO, TiO_2_ and SiO_2_, ZnO nanoparticles were the most cytotoxic to all three intestinal cell types. Different cellular responses among the three cell types owes to their different genetic landscape	[64]
Iron oxide										
Wen Zhang	2010	Caco-2 cells	100, 200 and 300 μg/ml from 5 to 45 min	Not reported (self-synthesized)	DLS and TEM	Not reported	Dose range not justified but adsorption kinetics taken into consideration	Not reported	Adsorption of hematite nanoparticles on Caco-2 cells is size dependent. Longer exposures induce tight junction disruption, and microvilli reorganization and detachment	[98]
Madhavi Kalive	2012	Caco-2 cells	1, 10 and 100 ppm from 5 to 28 days	Not reported (self-synthesized)	DLS, PdI and zeta potential in DI water and culture medium, ICP-MS	No standard dispersion protocol specified	Not reported	Not reported	Hematite nanoparticles potentially induce structural changes in the Caco-2 epithelium and the effects at cellular and genetic level are size-dependent	[66]

(alphabetical): *AAS* Atomic absorption spectroscopy, *BET* Brunauer-Emmett-Teller, *CB* Carbon black, *DLS* Dynamic light scattering, *DRIFTS* Diffuse reflectance infrared Fourier transform spectroscopy, *FTIR* Fourier transform infrared spectroscopy, *ICP-MS* Inductively-coupled plasma mass spectrometry, *ICP-OES* Inductively-coupled plasma optical emission spectrometry, *IGF-1* Insulin-like growth factor 1, *MET-1* Microbial ecosystem therapeutic-1, *NTA* Nanoparticle tracking analysis, *PdI* Polydispersity index, *SEM* Scanning electron microscopy, *SLD* Static light diffraction, *SSA* Specific surface area, *STNPs* Surface treated nanoparticles, *TEM* Transmission electron microscopy, *TEM-EDS* Transmission electron microscopy-energy dispersive spectroscopy, *XAS* X-ray absorption spectroscopy, *XPS* X-ray photoelectron spectroscopy, *XRD* X-ray diffraction, *XRF* X-ray fluorescence

The Sprague Dawley rat model and CD-1 (ICR) mouse model were used in 13 out of 19 of the in vivo studies reviewed (Table 3). In one case, an ex vivo animal model comprising of Peyer’s patches and ileum was used. Nanoparticles were delivered by gavage as dispersions in a food matrix.

**Table 3:** Overview of the key findings regarding the state of science in in vivo nanotoxicity testing of food-grade nanomaterials, categorized by nanomaterial type

First author	Year	Test system	Dose range	Nanomaterial grade	PCM characterization	Standardized dispersion and characterization	Dose range rationale	Dissolution biokinetics	Main conclusions from study	Ref
Titanium dioxide										
Jiangxue Wang	2007	CD-1 (ICR) mouse model	5 g/kg bw	Not reported	XRF analysis only	Not reported	Not reported	Not reported	TiO_2_ retained in liver, spleen, kidneys and lung tissues, suggesting uptake by gastrointestinal tract	[21]
Yanmei Duan	2010	CD-1 (ICR) female mouse model	62.5, 125 and 250 mg/kg bw	Not reported (self-synthesized)	XRD, ICP-MS analysis	Not reported	Not reported	Not reported	Intragastric TiO_2_ administration in mice damages homeostasis blood system and generates immune response resulting in disruption of liver function	[85]
Carolina M. Nogueira	2012	Bl 57/6 male mouse model	100 mg/kg bw	Commercially available for use in food, pharmaceuticals and cosmetics	DLS, XRD	No standard dispersion protocol specified	Not reported	Not reported	TiO_2_ micro and nanoparticles induce a Th1-mediated inflammatory response in the small intestine, especially ileum	[48]
Yun Wang	2013	Sprague Dawley male rat model	10, 50 and 200 mg/kg bw	Not reported	TEM, ICP-AES, XRD, FTIR, SSA by BET method, hydrodynamic size, zeta potential	No standard dispersion protocol specified	Intragastric doses selected based on the intake of dietary TiO_2_ particles in the UK	ICP-MS and ICP-OES to measure Ti content in tissues	Young rats seem more susceptible to TiO_2_ nanoparticle exposure, which can provoke reductive stress in the plasma of both young and old rats but through different mechanisms	[96]
Zhangjian Chen	2014	Sprague Dawley male rat model	10, 50 and 200 mg/kg bw/day for 30 days	Not reported	Previously characterized [96]	No standard dispersion protocol specified	Intragastric doses selected based on the intake of dietary TiO_2_ particles in the UK	Not reported	TiO_2_ nanoparticles induce DNA double strand breaks in rat bone marrow cells after repeated oral administration for 30 days. It might be practical to control the application of TiO_2_ nanoparticles as food additives	[62]
Roberta Tassinari	2014	Sprague Dawley rat model	1 and 2 mg/kg bw/day for 5 days	Not reported	TEM, SEM, ICP-MS	No standard dispersion protocol specified	Dose levels selected based on the available data on the effects of TiO_2_ nanomaterials	ICP-MS to measure Ti content in tissues	TiO_2_ nanoparticles target endocrine-active tissues at dose levels relevant to human dietary intake; with no observable general toxicity and limited tissue deposition and damage in spleen	[126]
Emilie Brun	2014	Peyer’s patches and regular ileum (ex vivo), mice model (in vivo)	12.5 mg/kg bw	Not reported (self-synthesized)	SSA by BET, XRD, TEM, agglomeration state (DLS), zeta potential, XAS	For ex vivo experiments, nanoparticle suspensions pulse sonicated at 28% amplitude– corresponding power measured using a calorimetric procedure [104]. Not reported for in vivo experiments	Dose level selected based on daily intake of TiO_2_ by US children	Not reported	TiO_2_ nanoparticles pass paracellularly through the regular intestinal epithelium by disrupting tight junctions and localize in tissues beneath these epithelial layers	[46]
Zhangjian Chen	2015	Sprague Dawley rat model	2, 10 and 50 mg/kg bw/day for 30 or 90 days	Not reported	TEM, ICP-AES, XRD, FTIR spectroscopy, SSA by BET, hydrodynamic diameter (DLS), zeta potential	No standard dispersion protocol specified	Intragastric doses for rats selected based on the daily oral intake of TiO_2_ nanoparticles for children under the age of 10 years in the US	Not reported	TiO_2_ nanoparticles alone or in combination with glucose induce liver, kidney and heart injuries as well as changes in white blood cells and red blood cells in young rats. Interactions between TiO_2_ nanoparticles and glucose was different in different body systems, leading to synergistic or antagonistic effects accordingly	[123]
Fashui Hong	2015	ICR male mice model	2.5, 5 and 10 mg/kg bw/day for 60 days	Not reported (self-synthesized)	TEM, XRD, SSA by BET, hydrodynamic diameter (DLS), zeta potential	No standard dispersion protocol specified	Dose levels were selected based on a report of the World Health Organization from 1969	Not reported	TiO_2_ nanoparticles cause testicular toxicity, reduced sperm production, and induced sperm lesions in a dose dependent manner. These effects are in close relation to reductions in daily food and water intake, biochemical dysfunctions and oxidative stress	[65]
Zhangjian Chen	2015	Sprague Dawley rat model	2, 10 and 50 mg/kg bw/day for 30 or 90 days	Not reported	TEM, ICP-AES, XRD, FTIR spectroscopy, SSA by BET, hydrodynamic diameter (DLS), zeta potential	No standard dispersion protocol specified	Intragastric doses for rats selected based on the daily oral intake of TiO_2_ nanoparticles for children under the age of 10 years in the US	Not reported	Long-term (90 days) daily ingestion of TiO_2_ nanoparticles can exert mild and temporary cardiovascular toxicity by reduction in heart rate and systolic blood pressure, and increase in diastolic blood pressure	[149]
Ismael M. Urrutia-Ortega	2016	BALB/c male mice model	5 mg/kg bw for 10 weeks	Food grade TiO_2_ (E171)	SEM, TEM, Raman spectroscopy, hydrodynamic diameter (NTA), zeta potential	No standard dispersion protocol specified	Intragastric doses justified based on collective exposure to TiO_2_ from nominal consumption estimates and other sources	Not reported	TiO_2_ E171 nanoparticles enhance tumor formation in the distal colon of chemical induced colitis-associated cancer (CAC) model of male BALB/c adult mice, marked by increase in CAC tumor progression markers.	[49]
Hanqing Chen	2017	CD-1 (ICR) male mouse model	2.5 mg/kg bw/day for 7 days	Not reported to be food-grade	TEM, hydrodynamic diameter (DLS), zeta potential	No standard dispersion protocol specified	Oral gavage doses justified based on estimated daily intake of TiO_2_ and SiO_2_, and recommendation of OECD for Ag [150]	Not reported	Ag nanoparticles cause colitis-like symptoms in intestinal tract, and changes in gut microbiome. SiO_2_ nanoparticles cause significant increase in proinflammatory cytokines and microbial species diversity. TiO_2_ nanoparticles did not induce obvious changes in GIT histology or gut microbiota composition	[124]
Maria G. Ammendolia	2017	Sprague Dawley rat model	1 and 2 mg/kg bw/day for 5 days	Not reported	TEM, SEM, hydrodynamic diameter (DLS), PdI. SSA and purity (provided by manufacturer)	No standard dispersion protocol specified	Dose levels selected based on the available data on the effects of TiO_2_ nanomaterials	ICP-MS to measure Ti in gut tissue	Higher dose of TiO_2_ nanoparticles in male rats causes increase in height and width of villus, and dose-related increase in density of goblet cells. No such effects are seen on female rats. TiO_2_ nanoparticles penetrate intestinal mucosa (suggested by ICP-MS data)	[125]
Fashui Hong	2017	ICR male mice model	1.25, 2.5 and 5 mg/kg bw/day for 9 months	Not reported (self-synthesized)	TEM, XRD, SSA by BET, hydrodynamic diameter (DLS) [151]	No standard dispersion protocol specified	Dose levels were selected based on a report of the National Institute for Occupational Safety & Health (NIOSH) from 2011 [152]	ICP-MS to measure Ti in gastric tissues	Long term exposure to nano TiO_2_ results in dysfunction of gastric secretion, inflammation, atrophy, and other lesions of gastric mucosa, which is closely associated with alterations of inflammation responding signal pathways in the stomach.	[100]
Sarah Bettini	2017	Adult male Wistar rat model	10 mg/kg bw/day for 7 days	Food grade TiO_2_ (E171)	TEM, TEM-EDX, XANES, hydrodynamic diameter, PdI, zeta potential	TiO_2_ products prepared following the generic Nanogenotox dispersion protocol [153]	Not reported	TEM-EDX analysis in liver and intestine, and NanoSIMS analysis in Peyer’s Patches	Intragastric food-grade TiO_2_ administration for one week impairs intestinal immune homeostasis through Th17-driven autoimmune complications. Chronic exposures correlating with the development of an inflammatory microenvironment, may initiate and promote expansion of preneoplastic lesions in the colon.	[50]
Silicon dioxide										
Hanqing Chen	2017	CD-1 (ICR) male mouse model	2.5 mg/kg bw/day for 7 days	Not reported to be food-grade	TEM, hydrodynamic diameter (DLS), zeta potential	No standard dispersion protocol specified	Oral gavage doses justified based on estimated daily intake of TiO_2_ and SiO_2_, and recommendation of OECD for Ag [150]	Not reported	Ag nanoparticles cause colitis-like symptoms in intestinal tract, and changes in gut microbiome. SiO_2_ nanoparticles cause significant increase in proinflammatory cytokines and microbial species diversity. TiO_2_ nanoparticles did not induce obvious changes in GIT histology or gut microbiota composition	[124]
Zinc oxide										
Vyom Sharma	2012	Swiss albino male mouse model	50 and 300 mg/kg bw	Not reported	DLS and TEM	No standard dispersion protocol specified	Followed OECD guidelines [154]	Not reported	Sub-acute oral exposure to ZnO nanoparticles leads to their accumulation in liver causing oxidative stress-mediated DNA damage and apoptosis	[81]
Surekha Pasupuleti	2012	Sprague Dawley rat model	5, 50, 300, 1000 and 2000 mg/kg bw	Not reported	DLS, zeta potential, SEM	Not reported	Followed OECD guidelines [155]	Not reported	Nano-sized ZnO exhibit toxic effects (increased AST and ALT serum levels, microscopic lesions in various organs) at lower doses in comparison to micron-sized ZnO	[79]
Miri Baek	2012	Sprague Dawley rat model	50, 300 and 2000 mg/kg bw	Not reported	XRD, SEM, TEM, zeta potential	No standard dispersion protocol specified	Not reported	ICP-AES to measure Zn content in tissues	ZnO nanoparticles accumulate in the form of zinc ions in the liver, kidney and lung irrespective of the gender or particle size. Excretion occurs via feces with higher rate of clearance for smaller particles	[117]
Yanli Wang	2014	Kunming male mice model		Not reported	XRD, TEM, XRF, hydrodynamic size (DLS) in water and cell culture medium, zeta potential	Not reported	Dose levels agreed with the European food additives standard and Chinese food additive standard	ICP-MS to measure Zn content in tissues	ZnO nanoparticles, in the presence of Vitamin C, induce significant changes in the TBIL (total bilirubin levels) and BUN (blood urea nitrogen) of liver and kidney, and trigger injury to the main organs	[63]

(alphabetical): *ALT* Alanine aminotransferase, *AST* Aspartate aminotransferase, *BET* Brunauer-Emmett-Teller, *DLS* Dynamic light scattering, *FTIR* Fourier transform infrared spectroscopy, *ICP-AES* Inductively-coupled plasma atomic emission spectrometry, *ICP-MS* Inductively-coupled plasma mass spectrometry, *NanoSIMS* Nanoscale secondary ion mass spectrometry, *NTA* Nanoparticle tracking analysis, *OECD* Organization for Economic Co-operation and Development, *SEM* Scanning electron microscopy, *SSA* Specific surface area, *TEM* Transmission electron microscopy, *XANES* X-ray absorption near edge structure, *XAS* X-ray absorption spectroscopy, *XPS* X-ray photoelectron spectroscopy, *XRD* X-ray diffraction, *XRF* X-ray fluorescence

#### Test ENM identity: Food or industrial grade?

**Fig. 3:**
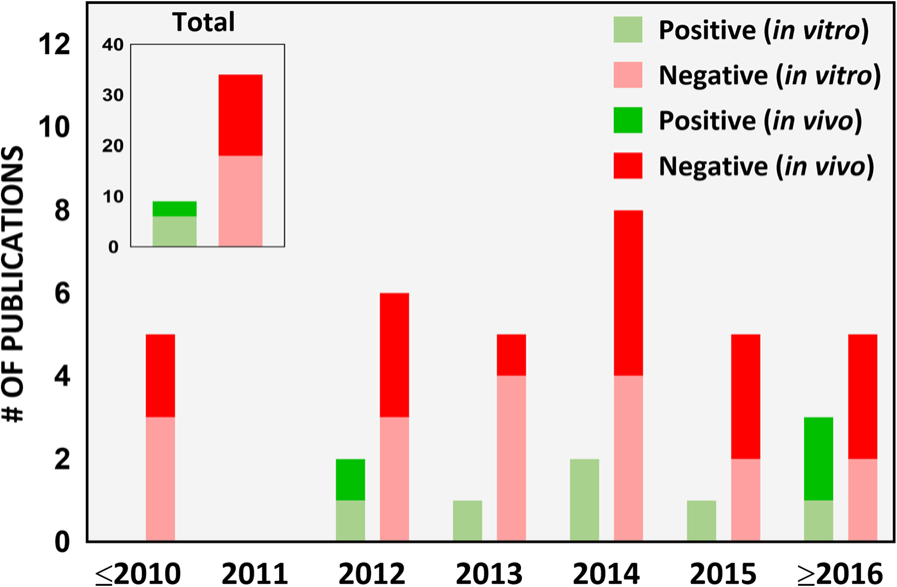
Food-grade nanomaterials. Number of scientific publications on ingested toxicity of nanomaterials from 2007 to 2017 using food-grade nanomaterials in the study

Table 2 summarizes the in vitro studies intending to assess toxicity of iENM, including any information in each study regarding the nanomaterial’s grade, nanomaterial characterization, consideration of dosimetry and physiological relevance, as well as the primary findings of each study. In our literature survey from 2007 to 2017, only 19% of the studies (8 out of 42) used food-grade nanomaterials to assess their toxicity on intestinal/gastric epithelial cells in an ingested nanoparticle exposure scenario (Fig. 3). Ingestion of food containing ENM is the primary exposure route of the GI tract to exogenous ENM. A lifecycle study of food grade SiO_2_ found that 10 out of 14 foods contained SiO_2_ of the same morphology and size as the pristine food grade SiO_2_ [14]. For 4 out of 14 foods; however, they may also have contained non-food grade SiO_2_. In this context, using food-grade ENM for toxicity testing is critical. Importantly, a small fraction of inhaled nanoparticles can be transferred to the GI tract through mucociliary escalator clearance mechanisms and swallowing [12]. This was demonstrated after an intratracheal instillation of a single dose of radiolabeled TiO_2_ NPs in Wistar-Kyoto female rats, where up to 5% of the instilled dose reached the GI tract after 24 h, which subsequently increased to 20% after 28 days [47]. Because workers and consumers could be exposed to a variety of nanoparticle types via inhalation, which are categorically far more diverse than food grade ENM, this may lead to GI tract exposure of a larger variety of nanoparticles. However, the dose and dose rate employed must be realistic and reflect the peculiarities of such ingestion through inhalation exposure pathway. Among in vivo studies, we found that 14 out of 16 studies did not report the grade of the test nanomaterial (Table 3). The study by Nogueira et al. [48], which used TiO_2_ commercially available for use in food, pharmaceuticals and cosmetics, reported that these ENM induced a Th-1-mediated inflammatory response in the small intestine, with more pronounced cytokine production in the ileum. Urrutia-Ortega et al. [49], which used food-grade TiO_2_ (E171), showed that TiO_2_ nanoparticles used as food additive can enhance tumor formation in the distal colon in a colitis-associated cancer (CAC) model of male BALB/c adult mice, accompanied by a marked by increase in CAC tumor progression markers (COX2, Ki67 and β-catenin). Bettini et al. further showed that chronic exposure to TiO_2_ E171 promoted ACF formation in normal mucosa, demonstrating the ability of food-grade TiO_2_ to promote the development of preneoplastic lesions in rats without pre-existing epithelial barrier injuries [50]. Such studies provide more direct evidence on safety concerns of the ENM added to food products, and further supports the relevance of testing these nanomaterials.

There is an abundance of nanotoxicology literature documenting the impact of variations in physico-chemical and morphological (PCM) properties of an ENM on toxicological outcomes. Differences in the primary particle size, surface area and chemistry, along with metal impurities, surface functionalization, or particle morphology, impact their behavior in and interaction with biological systems in vitro and in vivo [51]. The most compelling examples come from the ‘safe-by-design’ cases, where deliberate modifications in the surface chemistry, such as encapsulation of surfaces of ENM with amorphous silica [52, 53], or doping of ZnO with small amounts of Fe to suppress dissolution [54, 55], have a striking impact on the toxicity outcome. Food grade ENM are no exception. Toxicological findings on GI toxicity from industrial grade TiO_2_ (different size range, crystalline phase, and impurities) do not extend to food grade TiO_2_ and vice-versa. The same can be said for other iENM types.

Thus, when conducting toxicity assessment of ENM in the GI tract, it is important to use materials intended for or used in food, regardless of the test system. Several ingested nanoparticle toxicity studies used commercially available nanomaterials with no specification of the nanomaterials’ grade (Table 2 and Table 3) and its commercial use or application [56–64]. In other studies, tested nanomaterials were synthesized in the lab [46, 65–67]. Numerous studies examining the toxicity of TiO_2_ nanoparticles on intestinal cells reported use of photocatalytic TiO_2_ [68–70] rather than food grade/pigment TiO_2_ (Table 2), even though food grade TiO_2_ is the primary source of TiO_2_ in food products. Photocatalytic TiO_2_ has antimicrobial properties and is used in food contact materials such as food preparation surfaces, self-cleaning and de-polluting paints and microbial surfaces [71], which can act as a secondary source of TiO_2_ introduction into food products. There are significant differences between industrial and food grade TiO_2_ in terms of size, size distribution, specific surface area, surface properties and their agglomeration in aqueous phases, as discussed in detail by Dudefoi et al. [72]. For example, the primary particle sizes in photocatalytic P25 TiO_2_ were below 100 nm, whereas only 17–35% of the primary particles were under 100 nm in diameter in the food-grade TiO_2_ [73]. Yang et al. reported that cationic dyes adsorbed more readily to food grade TiO_2_ than P25 TiO_2_ [73], presumably due to the presence of phosphate groups on the surface of food grade TiO_2_ but not P25 TiO_2_ [72]. These differences in surface chemistry implies different potential for interaction with organics, proteins, and other micronutrients in the food matrix. In another scenario, where TiO_2_ used in sunscreens ends up being ingested (via swimming pool water, spoiled clothing in the workplace, or a child ingesting sunscreen by accident), surface treated TiO_2_ nanoparticles used commercially in sunscreens (such as T-Lite™) must be used for in vitro and/or in vivo studies [74]. Chen et al. [16] used nano TiO_2_ extracted from several chewing gums and found that its cytotoxicity was higher than that of commercially available P25 TiO_2_. At low concentrations of 350 ng/mL (100 ng/cm^2^), food grade/pigment TiO_2_ can also cause subtle changes in cell morphology, such as disruption of the brush border epithelium, but these concentrations did not acutely damage intestinal epithelium [26].

#### Food/product matrix effect

Selection of test ENM that are representative of the ingested exposure scenario and contained in the product that is actually ingested is critical for the relevance of a study. Using food grade variants instead of any available commercial forms of the test ENM will not only enable exposure scenario-relevant study designs but could also contribute towards reproducible observations across labs and more relevant toxicological outcomes [16, 73]. In other scenarios, such as assessing the hazard or risk of iENM resulting from ingestion of ENM from cosmetics and sunscreens, using food grade variants of those ENM would be of little utility. Instead, using the nanomaterials present in these cosmetic products lead to more relevant tests. Another critical factor to be considered for such tests is the matrix in which these iENM reside. When used in food and cosmetic formulations, ENM are immersed in a complex matrix of organic and inorganic additives, which interact with and become absorbed onto the surface of ENM, resulting in the formation of coronas with organic biomolecules such as proteins, lipids, and sugars. Such surface modifications may influence cellular uptake of ENM, their biomolecular recognition, dissolution behavior, and eventually their toxicity. The food matrix effect has been largely ignored in in vitro and many in vivo studies until very recently. Arguably, the food matrix is a bigger challenge to address properly in in vitro studies, but it is an important consideration [75]. In such cases, simulating exposure scenarios that closely resemble the process by which iENM are incorporated into or used in a food matrix, and variations in the food matrix itself, are essential. Furthermore, one should consider the complex journey that iENM undergo as they pass through the GI tract and the many PCM changes that they experience as a result of their transition through various compartments of the GI tract (e.g. exposure to different acidity, pH, digestive enzymes, food components, etc.). Exposing intestinal cells to pristine iENM makes unrealistically bold assumptions about representativeness of this test system. This aspect of the test methodology would benefit from more guidance, consensus documents, standardized protocols, and reference materials.

### Comprehensive PCM characterization in dry state

**Fig. 4:**
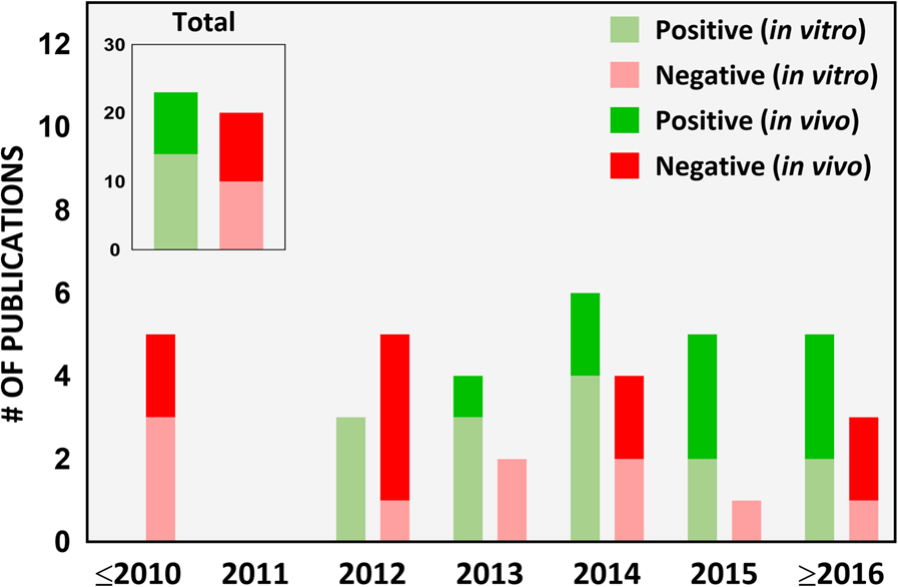
Physicochemical and morphological (PCM) characterization. Number of scientific publications on ingested toxicity of nanomaterials from 2007 to 2017 performing or referring to comprehensive PCM characterization of the nanomaterials used in the study

The physicochemical and morphological (PCM) properties play a pivotal role in determining the kinetics of nanoparticles, their dissolution and interaction with cells in cell culture medium and its impact on biological responses [76, 77]. It is widely accepted in the scientific community that PCM characterization of ENM are of paramount importance in order to correlate PCM properties with biological/toxicological responses [78]. As per the European Food Safety Authority (EFSA) guidelines, adequate characterization of ENM used in food products should include chemical composition, particle size/size distribution, physical form and morphology, particle and mass concentration, specific surface area, surface chemistry, surface charge, redox potential, and chemical reactivity/catalytic activity [7]. [see Additional file 1: Table S1] highlights some of the most important physicochemical properties of nanoparticles and common characterization methods. In our survey from 2007 to 2016, we identified several studies (Fig. 4), especially pertaining to iENM, which had minimal to no PCM characterization [21, 48, 56, 58, 69, 79–81]. Inadequate PCM characterization of iENM, in the dry state and in food matrices persists to this day.

Our literature review identified 53% of studies (23 out of 43) lacking adequate characterization of nanomaterials in the dry state (Fig. 4). We also observed that in vivo studies (10 out of 19) lacked more in comprehensive PCM characterization than in vitro studies (see Tables 2 and 3). To relate the potential toxicity of iENM determined from in vitro or in vivo studies to the specific features of iENM [82, 83], various metrics of their PCM properties should be evaluated in powder form and in biological matrices [84]. Such parameters may include among others size, morphology, mass, surface area, aspect ratio, charge, solubility and surface chemistry. There appears to be an increase in the number of studies with more adequate PCM characterization in more recent years. Before 2015, 45% (13 out of 29) of the studies performed adequate PCM characterization, which increased to 71% (10 out of 14) in 2015–2017; yet, 29% of those studies still had insufficient characterization. A good example of the importance of an adequate PCM characterization approach is that of Gerloff et al. [68], which enabled identification of distinctive toxicity of TiO_2_ anatase/rutile mixed phase on Caco-2 cells over pure anatase TiO_2_. However, in 20 out of 43 studies, little or no characterization was performed [66, 67, 85]. PCM characterization is indispensable in a mechanistic study investigating the biological effects of TiO_2_, SiO_2_ and ZnO nanoparticles on different cell lines intended to represent the intestinal epithelium [64] (although no single cell line can accomplish that), as the PCM properties of nanoparticles alone are capable of instigating unique biological responses [77, 86, 87]. We encourage future studies, especially in vivo, to implement comprehensive PCM characterization of iENM that exclusively use relevant food grade nanomaterials.

### Standardized nanoparticle dispersions and their characterization

ENM properties are typically measured in dry powder state (e.g. mass or surface area per volume) to compare biological responses to ENM exposure in terms of administered dose. These comparisons do not take into account particle-particle and particle to physiological fluid interactions in the liquid suspension [82, 88–90]. These interactions largely depend on the dispersion protocol, PCM characteristics of nanoparticles [91], and the properties of the suspending media (pH, protein content, ionic strength, etc.) [92]. For in vitro testing, ENM, which are normally agglomerated in nanopowder form, are dispersed in certain liquid medium, typically DI water. The dispersed ENM are then transferred into a physiologically relevant dispersion media, which are either cell culture media for in vitro studies or body fluids for in vivo studies [32]. The methods used to disperse ENM in physiologically relevant media for in vitro studies can have a substantial impact on the size, size distribution and the overall dispersion stability (re-agglomeration state). Additionally, the effective density of the agglomerates formed when ENM are dispersed in physiologically relevant media differs from the density of the raw material, at times by several fold, primarily due to the protein corona formation and intra-particle trapping of the media [34]. The effective density and agglomeration potential of the ENM affect their fate and transport in physiologically relevant media and impact the dissolution rate as well as available surface area for bio-interactions. The fate and transport of ENM in a media determines their settling rate, as well as other dose metrics such as delivered mass, surface number and particle number, each of which are discussed under dosimetry consideration. Consequently, in several in vitro studies, it has been shown that the agglomerates of nanoparticles exert different biological responses in comparison to well-dispersed nanoparticles [93–95]. Striking, but perhaps not surprising, is the finding that preparation of dispersions for nanotoxicity tests continues to be highly variable and non-reproducible across different labs, a practice that continues today.

The dispersion protocols used in the published literature of iENM to date are highly variable. In an in vivo study [96], TiO_2_ nanoparticles were dispersed in ultrapure water and ultrasonicated for 15 min before administering an intragastric dose. Another in vitro study by Zhao et al. [58] followed a protocol where TiO_2_ NP in methanol were bath sonicated for 30 min followed by diluting the stock solution to 10 μg/mL in complete cell culture medium, and then further bath sonicating the suspension for 10 min before cell culture treatment. Another set of in vitro studies examining toxicity of several nanoparticles in Caco-2 cells suspended all materials in serum-free media and bath sonicated for 7–10 min before adding the testing concentration to the cells [61, 68, 80]. McCracken et al. [59] and Tassinari et al. [97] both procured similar TiO_2_ (< 25 nm particle size, 35–65 m^2^/g surface area) from Sigma-Aldrich but the former pulse sonicated 1 mg/mL nanoparticle suspensions in 1 × PBS for 1 s on/1 s off cycle for approximately 15 s, whereas the latter sonicated 2 mg/mL suspensions with a probe sonicator for 15 min. In a different set of studies examining toxicity and cellular responses of intestinal cells exposed to various nanomaterials, nanomaterial suspensions in DI water were either synthesized in-house or purchased directly from the manufacturer, and no further sonication was performed [56, 63, 79, 98, 99]. Among in vivo studies with TiO_2_ nanoparticles, 0.5% hydropropylmethylcellulose (HPMC) was used as a suspending agent [21, 65, 85, 100]. Even though Teubl et al. [70], in a study of the buccal mucosa as a possible route for TiO_2_ nanoparticle uptake, ultrasonicated nanoparticle suspensions for 1-24 h to evaluate the optimal duration at which the suspensions had the lowest mean particle sizes, the type and make of the sonicator, as well as the sonication parameters were not reported. Clearly, standard protocols for dispersing powdered nanomaterials in deionized (DI) water and cell culture medium [101] need to be employed and precisely reported to allow direct comparisons across studies and independent replication of the results. Standardized protocols will enable comparisons and evaluations of in vitro as well as in vivo studies across labs. Furthermore, studies attempting to understand the effects of surface-coated or surface-treated nanoparticles [74] should avoid extensive sonication as it can cause removal of surface coating through large, but very localized, forces produced by cavitation [102, 103], which in turn can potentially alter biological responses to each type of treated nanoparticles.

Highly reproducible and standardized methodologies for nanoparticle dispersion, along with fate and transport modeling (discussed in greater detail in the next section), have been developed over the past few years, with the first papers on this topic appearing in 2012. Taurozzi and Hackley [104] published a detailed study on preparing standardized nanoparticle dispersions and reporting on the precise conditions so that the dispersion protocol is reproducible among other labs. Fully validated and transferable dispersion and transport modeling protocols are now available for common cell lines and ENM in the context of inhalation nanotoxicology, and they can be adopted for iENM [31–33, 105]. Yet, the practice has not changed significantly across the broader scientific community, and as a result, progress has been slow. The impact of such practices has not been properly documented among in vitro nanotoxicology studies of iENM, but based on our literature review, we hypothesize that it could be significant. Some important factors related to sonication conditions, for which there is strong evidence in the published literature, include variation in size distribution, dispersion stability, ion release/concentration, generation of free radicals and non-radical oxidants such as hydrogen peroxide during the sonication process that get transferred to the cell culture medium, and other modifications to nanoparticle surface properties. Spurious oxidant production may negatively affect assay performance and/or confound in vitro results.

### Dose range, rationale and dosimetry considerations

#### Dose range and rationale

The importance of dose rate and dose range in nanotoxicology testing has been documented in at least two recent nanotoxicology studies [106, 107]. The same logic holds true for testing iENM. Establishing a dose range that is realistic and physiologically relevant should take into consideration real-world ingestion (and inhalation) exposures, their frequency and other important features, such as administration of iENM in complex and diverse food matrixes. The challenges of selecting a defensible dose range is greatest in in vitro nanotoxicology of ingested ENM, for the simple reason that cell monocultures or co-cultures in a plate represent a very different environment compared to that of cells within an organ. Assessment of a relevant dose or dose range requires extrapolation of estimated human daily intake or exposure data for a specific ENM to equivalent in vitro doses for a relevant cell culture model [108]. As an example, if an individual ingests 1 mg of TiO_2_ through chewing gum, the amount of dose and time for which the buccal cavity cells are exposed will be different from the intestinal epithelial cells. Even if either cell type ends up being exposed to the same amount, the surface area of each tissue will change the amount the cells are exposed to per unit surface area. Although there have been several toxicity studies where in vitro doses (μg/mL) are converted to equivalent in vivo (mg/kg) doses for studies in mice, any attempt to extrapolate published human exposure values to equivalent in vitro ingested doses in nanotoxicology has not been reported so far.

**Fig. 5:**
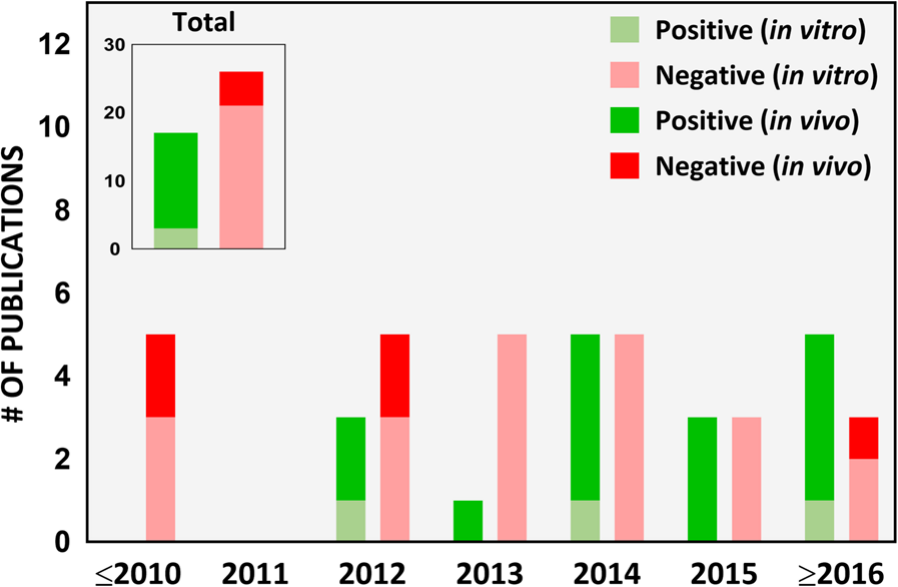
Dose range rationale. Number of scientific publications on ingested toxicity of nanomaterials from 2007 to 2017 which considered realistic exposure doses in the study

In our survey of the literature from 2007 to 2017, we found that 40% (17 out of 42) of the iENM toxicity studies provided a dose range rationale, of which 14 were in vivo studies (Fig. 5). In in vitro studies (Table 2), the dose range, dose metric and time points varied across the study. For example, in studies assessing cytotoxicity of TiO_2_ nanoparticles on Caco-2 cells, groups have reported the following: 100 and 1000 μg/mL as acute and chronic exposure doses, respectively [56]; 0.35 to 35 μg/mL dose range [26]; 20 and 80 μg/cm^2^ for 4 and 24 h exposures [68, 80], respectively; 1 to 20 μg/cm^2^ for 6 and 24 h exposures [60]; 0 to 200 μg/mL for 24 h exposures [16, 69]; 0 to 500 μg/mL for 48 h exposures [46]; 50 and 200 μg/mL for 24 h exposures [99]; and 1 mg/L for 0 h, 2 h, 4 h, 6 h, 8 h and 24 h exposures [109]. None of these reports provided a rationale for the indicated dose amounts. It is also not clear how any of these doses compared to the tissue doses (small intestines in this case) in vivo in animal studies and/or in humans. Although in most studies, Caco-2 cells were treated with nanoparticles only after verifying the formation of an intact epithelium by following a standard procedure of allowing the cells to grow for 19–21 days, measuring TEER (Trans-epithelial electrical resistance) values and expression of tight junction proteins [42–44]. In other studies, cells were treated within 12 h (overnight) to 4 days of cell growth without confirmation of intact intestinal epithelium [60, 69, 80, 109]. In an in vitro study examining the effects of surface-treated TiO_2_ nanoparticles widely used in sunscreens on Caco-2 cells, Fisichella et al. [74] deliberately chose the dose range to be higher than predicted environmental concentrations (10 to 100 μg/mL versus an expected 0.0007 to 0.016 μg/mL) under the assumption of a possible increase in the local environment, such as a child ingesting sunscreen by accident.

**Fig. 6:**
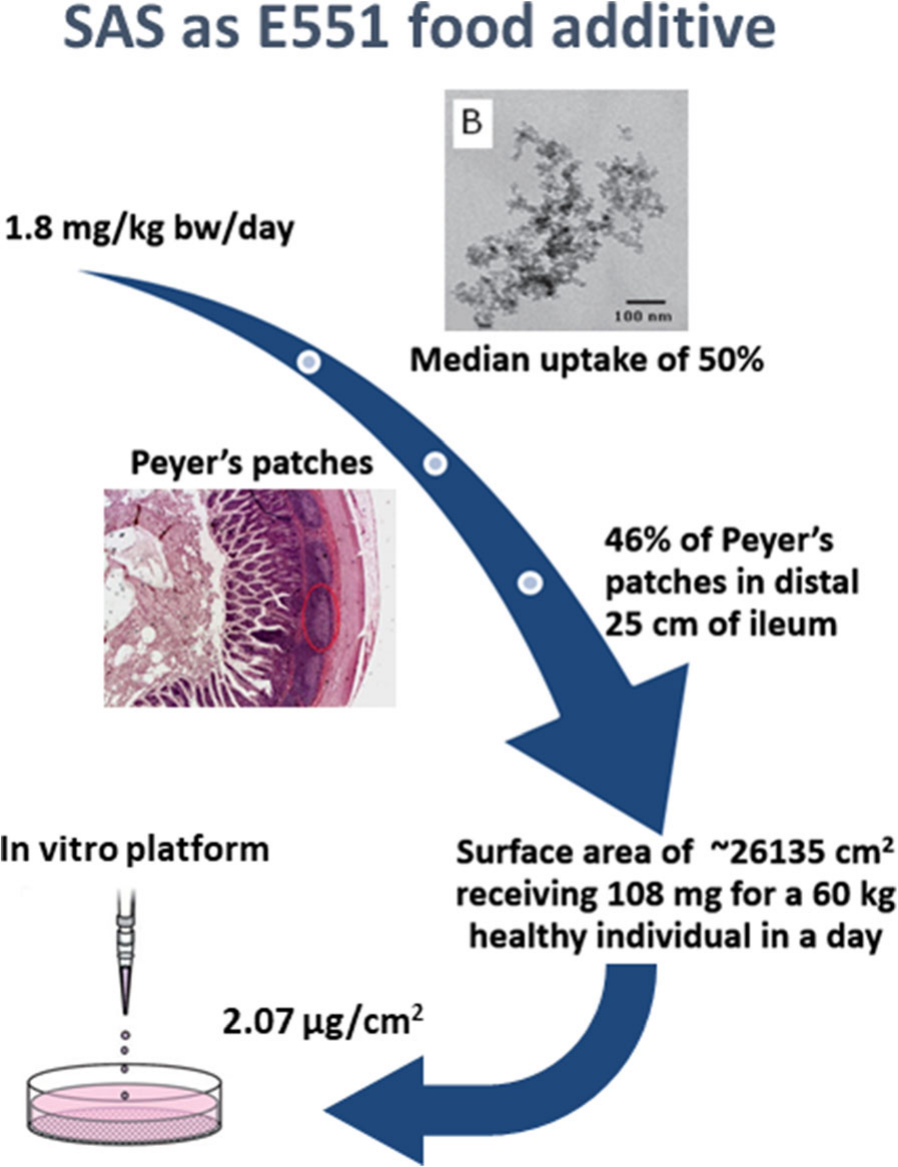
In vitro dose determination (extrapolation concept). In-human to in vitro dose extrapolation of E551 synthetic amorphous silica (SAS) for using realistic dose ranges in an in vitro cytotoxicity model

The dose ranges used in existing in vitro iENM toxicity studies differ widely between groups and are not validated based on in vivo or in-human daily intake or exposure data (Table 2). More importantly, even though the estimated human daily intake differs for each ENM, the same dose range is used for different nanomaterials in multi-nanomaterial in vitro studies [61, 64, 80]. However, the use of high doses may be desirable for comparative assessment and hazard ranking but is of limited utility if the study relies on a single unrealistically high dose or when it lacks a dose range and dose-response analysis. These dose issues continue to be prevalent in in vitro nanotoxicology of iENM. On the other hand, we found that dose ranges used in most in vivo iENM toxicity studies (14 out of 19) were well justified based on OECD guidelines (Organization for Economic Co-operation and Development) or estimated daily dietary intake of iENM (Table 3). For in vitro iENM toxicity studies, we recommend using a mathematical approach for calculation of nominal doses based on published estimated daily dietary intake values to equivalent in vitro doses, from which a range of doses can then be selected. Such an extrapolation, although not reported so far, would require consideration of several factors such as estimated daily intake (mg/kg bw/day), exposure variability (also known as median exposure dose), exposure/dose at the tissue site, estimated surface area of the exposure site (cm^2^), and other biokinetic considerations. Figure 6 illustrates conceptually the process of calculating a nominal equivalent in vitro dose for Caco-2 cells.

#### Dosimetry consideration

**Fig. 7:**
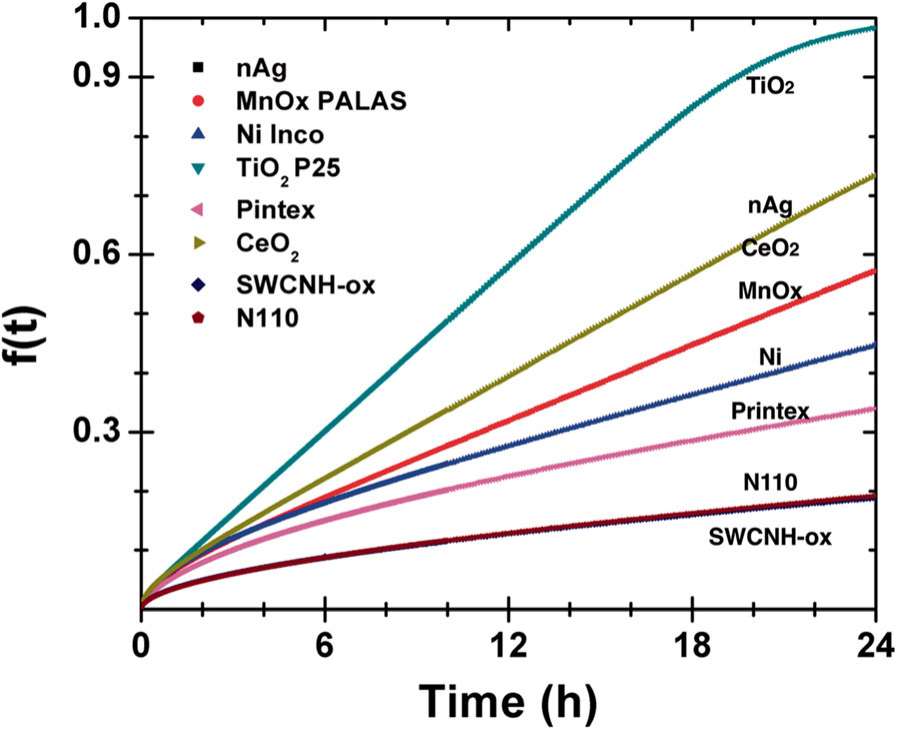
VCM-ISDD model-based calculations for nm delivered dose for different ENM formulation in RPMI + 10% FBS medium. Reproduced in parts with permission from Pal et al. [32]

An in vitro cell culture system, which is usually a simplistic representation of a complex biological system, is a valuable tool to study cell biological, physiological and pathological processes under stress. However, as a platform, in vitro mono- or bi/tri-cultures have their own problems and limitations. One such challenge relates to delivery of nanomaterials in dispersions. The dynamics of the in vitro system can have a profound effect on the outcome and/or the interpretation of the results. Most in vitro studies report administered doses in terms of either an initial mass concentration or of a total administered mass, but it is of great importance to consider the actual dose delivered to cells over time. The in vitro dosimetry concept, and its importance and applicability, has been discussed in much detail in several recent and important papers [88, 110, 111]. The delivered dose (in the form of mass or ions) is the fraction of the administered dose that ends up depositing on the cell monolayer in an in vitro system in a given time, which eventually interacts with the cells to trigger a biological response. Dose delivered is largely dependent on the intrinsic PCM properties of the suspended nanomaterial, the extrinsic properties of culture media in which the nanomaterial is suspended, and the time course of exposure. Presence of larger agglomerates and effective density also impact the dose deposition kinetics [33, 34, 112], which emphasizes the significance of standard dispersion protocols and their characterization. Furthermore, presence of a sticky mucus layer, such as in co-cultures of epithelial (Caco-2/C2BBe1) and goblet cell lines (HT29-MTX), could drastically change the dose deposition and uptake kinetics in comparison to a monoculture of Caco-2/C2BBe1, which is devoid of mucus. Thus, any applied dose should be representative of a relevant dose experienced by the specific cell culture system under a realistic exposure scenario. In other words, delivered dose (or the dose range) is mainly dependent on the cell type used in the study, and the administered dose should take into consideration the in vitro dosimetry. As illustrated in Table 2, our evaluation affirms that, to our knowledge, since 2007, no in vitro study in the realm of iENM toxicity considered dosimetry and its implications, which can potentially have a profound impact on the outcome and/or the interpretation of results. In an assessment of the impact of dosimetry, Pal et al. [32] found that after taking dosimetry into consideration, the slopes of administered/delivered dose-response relationships changed 1:4.94 times and were ENM-dependent, which significantly changed the toxicological ranking of engineered nanomaterials. Moreover, the resultant overall relative ranking of ENM intrinsic toxicity matched the in vivo inflammation data much better (Fig. 7). With this in mind, an in vitro cell culture model is of great utility if it closely resembles or validates the in vivo conditions [113]. Future in vitro iENM toxicity studies should consider better modeling of exposures and equivalency that are relevant between exposure scenarios and in vitro dosimetry.

### Dissolution kinetics

**Fig. 8:**
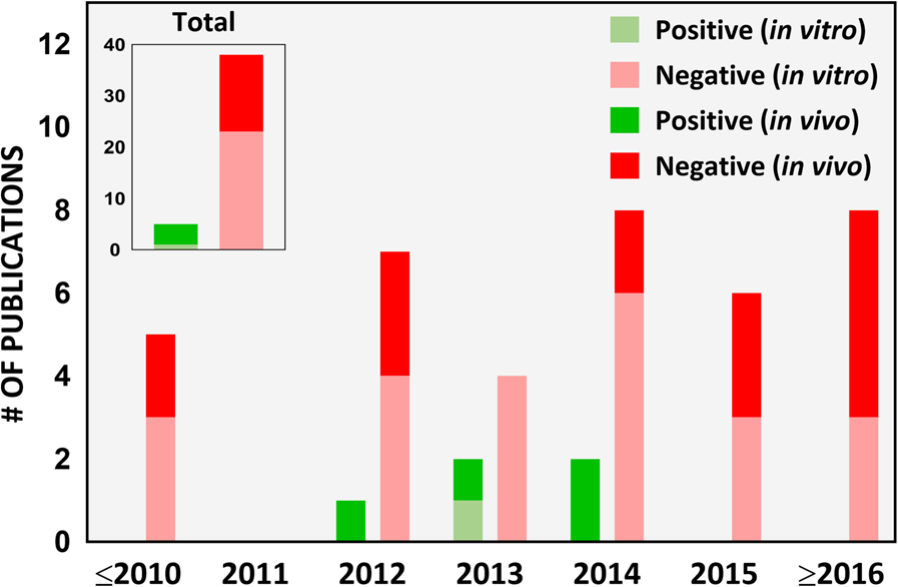
Dissolution biokinetics. Number of scientific publications on ingested toxicity of nanomaterials from 2007 to 2017 which considered nanomaterial biokinetics and dissolution in the study

The majority of nanoparticle toxicity studies require a dosing protocol in which the test material is required to be in a liquid phase (culture media), where the term “dispersion” instead of “solution” is used. Dissolution, in the case of nanomaterials, denotes release of ions or molecules from the surface of a nanomaterial and their distribution throughout the available liquid volume as a result of entropy [114]. Although, there is sizeable literature on dissolution and biodurability of natural and synthetic micro-sized particles and fibers, studies of iENM toxicity lack dissolution evaluation of these nanomaterials (Fig. 8) [59, 115]. The dissolution of nanoparticles in a culture media is largely driven by the concentration gradient that exists between the surface of NPs and the culture media. This, in turn, depends on the intrinsic PCM properties of nanoparticles, which include particle size, composition, shape, crystallinity, surface area and modification, and dispersion state of the nanoparticles. It also depends on the extrinsic properties of the culture media in which the NPs are dispersed, which includes parameters such as pH, ionic strength, constituent solvated molecules, temperature, ion concentration and availability of constituents to form complexes with released ions. This results in different dissolution rates for the same nanoparticles in different culture media with different order kinetics [116]. Such differences in dissolution rates necessitates its consideration when reporting the biological effects of nanoparticles. In addition, the dissolution state of nanoparticles (particulate form or dissolved state) in a dispersion medium is a key component of the dynamic process that determines their uptake pathway, mechanism of toxicity, and the biological compartment in which the NP will have highest potential impact [116]. It has been shown through in vivo studies that even when no nanoparticles could be seen in TEM images, accumulation of nanoparticles was evident on ICP-MS analysis, implying an ionized fate in the cells or tissues [96, 117]. Ionization is important in driving another phenomenon, tissue redistribution, translocation and formation of new nanoparticle species with different chemical composition (e.g., as phosphates, oxalates or carbonates) at a distal site [118]. This phenomenon has been documented well for cerium oxide nanoparticles (Ce^3+^ in Ce_2_O_3_ or Ce^4+^ in CeO_2_), where surface Ce^3+^ sites, the main driver of toxicity, become complexed with phosphate to form cerium phosphate (CePO_4_) completely reverts their toxicity and stimulates growth [119–121].

Dissolution of iENM in relevant GI tract media continues to be lacking in the literature. Only one out of 24 in vitro studies in the realm of iENM toxicity research addressed dissolution kinetics and its biological relevance (Table 2 and Fig. 8). Angelis et al. [60] in an in vitro study have shown significantly different dissolution kinetics of ZnO nanoparticles in serum or serum-free culture media, which drastically influences the cytotoxicity of these nanoparticles in Caco-2 cells. They showed that in serum-free media, toxicity of ZnO NP at lower concentrations was predominantly due to their dissolution into Zn^2+^ ions, whereas at higher concentrations it was caused by both ZnO nanoparticles and Zn^2+^ ions. It is, therefore crucial to consider the dissolution kinetics of nanoparticles in relevant cell culture media and its impact to in vitro studies.

Among in vivo studies, only 21% (4 out of 19) of the studies from 2007 to 2017 addressed dissolution kinetics (Fig. 8). Most of those in vivo studies measured the ionic forms of respective nanomaterials in various tissues [63, 96, 97, 117] (Table 3**)**. However, it is also essential to determine the dissolution kinetics, biodurability and biopersistance of nanomaterials in digestive fluids where nanoparticles interact with several complex fluids of varying pH, ionic strength and enzymatic activity during their course traveling through the gastrointestinal tract.

### Summary of toxicological endpoints and outcomes

Among in vitro studies, the effects of iENM on cell proliferation, cellular energetics (WST-1, WST-8, live/dead kit, CellTiter-Glo, XTT, MTT and NRU assay), membrane damage (LDH and Trypan blue assay), apoptosis initiation (Annexin V-FITC and monodansylcadaverine staining), necrosis (Sytox red staining), DNA damage (Fpg-modified comet assay), morphology (electron microscopy), barrier permeability (Dextran-FITC transport), reactive oxygen species generation (electron paramagnetic resonance, total glutathione content, DCFH-DA assay), proinflammatory and inflammatory cytokine release (ELISA), and gene expression (qRT-PCR) have been explored. Ion release from test nanomaterial and their subcellular location of accumulation has also been investigated using fluorescent labelling of Zn^2+^ ions [63]. The effects of iENM (e.g. TiO_2_) on in vitro models of gut microbiome has also been explored by monitoring gas production (gas chromatography), analysing fatty acid production (fatty acid methyl ester analysis), and microbiome diversity (16S rRNA 454 pyrosequencing).

Upon exposure to iENM, in vivo studies have determined the coefficients of liver, kidneys, stomach and spleen, biochemical analysis of the blood for biomarkers of liver, kidney, cardiac, thyroid and reproductive function, histopathological and ICP-MS/ICP-AES analysis of the tissues, hematological parameters, cytokine release and inflammatory cells quantification in GI tract segments, semen evaluation (sperm count, motility and % abnormal sperms, biochemical assay of enzyme activities and oxidative stress) for testicular toxicity, heart rate and blood pressure, tumor progression biomarkers (COX2, β-catenin and Ki67) and IL-1β, IL-2, IL-6, TNF-α, IFN-γ, IL-8, IL-10, IL-17 and GM-CSF levels in colon tissue, gut microbiome composition (16S rRNA by 454 pyrosequencing), Ti concentration in tissues (confocal microscopy, micro X-ray fluorescence imaging and NanoSIMS imaging), intestinal permeability (^51^Cr-EDTA radioactivity), and formation of aberrant crypts (preneoplastic lesions) using Bird’s procedure [122]. Table 4 provides an extensive summary of various toxicological endpoints and respective assays used in in vitro and in vivo iENM toxicity literature as well as the studies of the gut microbiome.

**Table 4:** Toxicological endpoints used and/or recommended in in vitro and in vivo iENM toxicity investigations, and studies of the gut microbiome

Endpoint	Used and/or recommended assays or procedures	References
In vitro		
Nanoparticle ion release and accumulation location	Fluorescent labeling of ions [156], ICP-MS	[63, 129]
Cell proliferation	Cell count using hemocytometer	[128]
Cellular energetics	WST-1, WST-8, live/dead kit, CellTiter-Glo, XTT, MTS, MTT, NRU, *Prestoblue* assay	[46, 69, 127]
ROS generation	Electron paramagnetic resonance, total glutathione content, DCFH-DA assay	[63, 64, 68, 127]
Cell membrane damage	LDH and trypan blue assay	[59]
Apoptosis initiation	Annexin V-FITC, monodansylcadaverine staining	[59, 64]
Necrosis	Sytox red and propidium iodide (PI) staining	[59, 63]
Pro-inflammatory and inflammatory cytokine release	ELISA, Wester blotting	[129]
DNA damage	Fpg-modified comet assay	[62, 68]
Brush border morphology	Immunocytochemistry, electron microscopy (TEM and SEM)	[66]
Barrier integrity	Trans-epithelial electrical resistance measurement	[46, 128]
Barrier permeability	Dextran-FITC and Lucifer yellow transport	[46, 128]
Gene expression	qRT-PCR	[46, 64, 66, 68]
In vivo		
Coefficients of organs	Ratio of tissue (wet weight) to body weight	[21, 85]
Changes in tissues	Histopathological evaluation	[48, 123, 149]
Testicular toxicity	Sperm count, motility and % abnormal sperms	[65]
Tissue accumulation	ICMP-MS or ICP-AES	[100]
Reductive stress	GSH/GSSG ratio in plasma	[96]
Tissue function	Blood biochemical and hematological analysis	[123, 149]
Inflammatory cells quantification in blood and the GI segment of interest	Flow cytometry, *imaging flow cytometry*^a^	[48, 50]
Apoptosis in the GI segment of interest	TUNEL assay	[81, 125]
Cytokine release in blood and the GI segment of interest	ELISA (IL-1β, IL-2, IL-6, TNF-α, IFN-γ, IL-8, IL-10, IL-17 and GM-CSF), Western blotting	[48, 49]
Tumor progression biomarkers in colon tissue	Immunohistochemistry (COX2, β-catenin and Ki67), *ELISA*^a^*, Western blotting*^a^	[49]
Intestinal permeability	^51^Cr-EDTA radioactivity	[50]
Aberrant crypts formation in the GI tract	Bird’s procedure [122]	[50]
Local tissue concentration	Micro X-ray fluorescence, NanoSIMS imaging	[46, 50]
Gut microbiome composition	16S rRNA pyrosequencing, *Shotgun metagenomic sequencing*^a^, *Microbial transcriptomics*^a^	[124]
Gut microbiome models		
Gas production	Gas chromatography	[45]
Fatty acid production	Fatty acid methyl ester analysis	[45]
Microbiome diversity	16S rRNA 454 pyrosequencing, *Shotgun metagenomic sequencing*^a^, *Microbial transcriptomics*^a^	[45, 124]

^a^Recommended assays or procedures – not used so far in the iENM toxicity literature

#### Titanium dioxide

Titanium dioxide was the most studied material in iENM toxicity research from 2007 to 2017, comprising 18 out of 24 in vitro studies and 14 out of 18 in vivo studies. Among in vitro literature, we observed contrasting results between similarly designed studies. Gerloff et al. [80] reported that 24 h exposure to 80 μg/cm^2^ TiO_2_ nanoparticles exerted cytotoxic effects on Caco-2 cells while no such effects were seen by Koeneman et al. [56] on Caco-2 cells at similar concentrations for up to 72 h. In a follow-up study, Gerloff et al. further highlighted the distinctive toxicity of rutile/anatase mixed TiO_2_ on Caco-2 cells but other studies reported no such effects [59, 60, 64]. However, Tada-Oikawa et al. [69] reported that 72 h exposure to anatase (50 nm) TiO_2_ nanoparticles reduced cellular viability of Caco-2 cells in a dose-dependent manner and induced proinflammatory response documented by increased levels of IL-1β and IL-8. Such inconsistencies among similar studies in the in vitro iENM toxicity literature could be attributed to the factors discussed in the previous sections of the review. Apart from cytotoxic effects, Brun et al. [46] reported possible translocation of TiO_2_ nanoparticles through regular epithelium of the ileum and Peyer’s patches, and Faust et al. [26] reported disruption of brush border morphology by nanoparticles-containing food-grade TiO_2_. Brun et al. further reported much higher accumulation of TiO_2_ nanoparticles in Goblet cells and M-cells in comparison to enterocytes. The accumulated TiO_2_ nanoparticles further induced tight junction remodeling by inducing deregulation of genes encoding for proteins involved in epithelial structure maintenance [46].

Persistence of TiO_2_ nanoparticles in specialized gut cells could possibly induce chronic damage. Indeed, this has been demonstrated by several long-term in vivo ingested exposure studies. An early single high dose study by Wang et al. demonstrated uptake of TiO_2_ nanoparticles through the GI tract and their retention in liver, spleen, kidneys and lung tissues in CD-1 (ICR) mouse [21]. TiO_2_ nanoparticles induced lung, kidney and heart injuries as well as changes in red and white blood cell count in Sprague Dawley rats in a dose, time and gender-dependent manner after a 90 day exposure to 0–50 mg/kg bw/day [123]. Reduction in sperm production and sperm lesions were induced in ICR male mouse in a dose-dependent manner upon exposure to 0–10 mg/kg bw/day for 60 days [65]. A 10 week exposure to TiO_2_ E171 for 5 mg/kg bw for 5 days/week enhanced tumor formation in the distal colon of chemical induced colitis-associated cancer (CAC) model of male BALB/c adult mice, marked by increase in CAC tumor progression markers in BALB/c male mice model [49]. Such colitis-like symptoms were not observed in CD-1 (ICR) male mouse after a 7-day exposure to 2.5 mg/kg bw/day of TiO_2_ nanoparticles [124] but Bettini et al. demonstrated that exposure to 10 mg/kg bw/day of food-grade TiO_2_ for the same time period impaired intestinal immune homeostasis through Th17-driven autoimmune complications in adult male Wistar rats [50], which was also observed in a similar study by Nogueira et al. after a single dose exposure TiO_2_ micro and nanoparticles commercially used in food products [48]. Bettini et al. further demonstrated that a 100 day exposure to food-grade TiO_2_ correlated with development of an inflammatory microenvironment, which promoted and could potentially initiate preneoplastic lesions in the colon [50]. A 9-month long exposure to nano TiO_2_ at 0–5 mg/kg bw/day also resulted in dysfunction of gastric secretion, inflammation, atrophy, and other lesions of gastric mucosa in ICR male mouse [100]. Interestingly, we noted that acute studies, in contrast to chronic ingested exposure studies, were much more likely to conclude with no observable toxic effects of TiO_2_ nanoparticles [46, 124–126].

#### Silicon dioxide, zinc oxide and iron oxide

In the past decade, a total of 7, 9 and 2 studies were published in regard to ingested exposure to SiO_2_, ZnO and Fe_2_O_3_ nanoparticles, respectively. Pertaining to the safety assessment of ingested SiO_2_ nanoparticles, all studies, except one, were conducted in vitro and there was limited agreement between them. Gerloff et al. in two studies [80, 127] and a study by Gehrke et al. [57] have shown that 24 h exposure to 80 μg/cm^2^ SiO_2_ nanoparticles induced cytotoxic effects, DNA damage and glutathione depletion in Caco-2 cells, and 24 h exposure to ~ 150 μg/cm^2^ SiO_2_ nanoparticles stimulated HT29 cells proliferation, interfered with glutathione biosynthesis and the toxicity was found to be dependent on concentration, size and FCS (fetal calf serum) content of the culture medium, respectively. On the other hand, SiO_2_ nanoparticles have been reported to be relatively safe and exhibited no/minimal toxic effects after 24 h exposure to C2BBe1 cells at 10 μg/cm^2^ [59], 24 h exposure to GES-1 and Caco-2 cells up to 100 μg/ml of food additive silica [128] and 12 h exposure to three intestinal cell lines (DLD-1, SW480 and NCM 460) at 1000 μM [64]. No overall toxicity beyond production of pro-inflammatory cytokines (IL-1β, IL-6 and TNFα) was observed when male CD-1 (ICR) mouse were administered 2.5 mg/kg bw/day SiO_2_ nanoparticles for 7 days but interestingly, microbiome analysis demonstrated increased microbial species diversity with an obvious increase in the genus *Lactobacillus* [124].

With regards to ingested ZnO nanoparticles, 5 studies were conducted in vitro, 3 in vivo and one study had both in vitro and in vivo aspects. All in vitro studies reported mild to significant toxic effects on different intestinal cell lines (Caco-2, C2BBe1, GES-1, DLD-1, SW480, NCM 460) when exposed to ZnO nanoparticles alone [59, 64, 80, 127, 129] or in combination with Vitamin C [63]. In vivo studies reported possible accumulation of ZnO nanoparticles (more likely in their ionic form) in the liver, lung and kidney with the smaller particles clearing from the body, primarily via feces, more rapidly than the larger ones [130]. A single dose of 5 to 2000 mg/kg bw to Sprague Dawley rats resulted in increased serum levels of ALT (alanine aminotransferase) and APT (aspartate aminotransferase), and microscopic lesions in liver, pancreas, heart and stomach at lower doses after 14 days [79]. Moreover, a 14-day consecutive exposure at 300 mg/kg bw/day to male Swiss albino mice also elevated serum ALT and alkaline phosphatase (ALP) levels, induced oxidative stress-mediated DNA damage and apoptosis – leading to pathological lesions in the liver [81].

In the past decade, only 2 in vitro studies investigated the effects of Fe_2_O_3_ on intestinal epithelial cells. The studies demonstrated size-independent adsorption of hematite nanoparticles on Caco-2 cells, which triggered dynamic reorganization of the brush border epithelium, disruption of tight junctions, drop in TEER, and differential expression of tight junctions-maintaining genes [66, 67].

To summarize, lack of in vitro and in vivo studies pertaining to ingestion of iron oxide nanoparticles, inconsistencies among similarly designed in vitro iENM toxicity studies of TiO_2_ and SiO_2_, and lack of in vivo studies in case of SiO_2_ are sizeable knowledge gaps in the safety assessment of iENM. Future studies, especially in vitro, should be mindful to not propagate similar methodological issues discussed earlier and inconsistent findings.

### Other factors

In addition to the factors discussed above, we would like to emphasize some additional factors that influence cytotoxicity of iENM, in particular co-culture cell models, consideration of the food matrix, cascade transformation of iENM as they go through the GI tract, and investigation of other endpoints beyond cell injury. The most common in vitro model employed in iENM toxicity studies in the past decade utilized Caco-2 monocultures (Table 2), differentiation of which is physiologically similar to the enterocytes of the small intestine, in vivo. While this may be a reasonable choice for many situations, the small intestinal epithelium is much more complex, which needs to be more accurately emulated in the in vitro test systems. The intestinal mucosa is protected by a layer of mucus secreted by both goblet cells and submucosal glands [131], and in some areas such as Peyer’s patches, they are also associated with the lymphoid tissue, which provides continuous antigenic surveillance of the intestinal contents. It is therefore more appropriate to use co-culture or tri-culture systems representative of the mucus-secreting or lymphoid-associated intestinal epithelium, which now have become available [46, 132]. Additionally, food-grade nanomaterials are ingested along with the food they are added to, which proposes the need to consider ENM-food interactions and their passage through the GI tract before exposing the in vitro cultures representative of the intestinal epithelium [75, 133–135]. Consideration of the passage through the GI tract will not be necessary if the in vitro test system is representative of the buccal mucosa. It has also been shown that chitosan nanoparticles enhance absorption and bioavailability of certain compounds [4, 136, 137]. iENM are no different and their effect on nutrient, and especially micronutrient absorption and bioavailability, need to be explored. Furthermore, small and large intestine are home to various microorganisms, which play an important role in human health [138–140]. In fact, changes in diet alone can cause rapid transformations in the activity and structure of the gut microbiota [141], and so can ingested iENM. It is therefore, not only appropriate, but critical to consider the effects of iENM on the gut microbiome in humans, as well as on in vivo or in vitro models representative of the colon [142, 143]. More importantly, humans are the ultimate consumer of iENM. With minor exceptions, human studies that investigate the links between iENM ingestion, diet, GI health, and human health in general, are currently lacking, and deserving of studying.

## Conclusion

In this review, we evaluated iENM toxicity literature over the last decade with the objectives of identifying best practices and recommending more relevant in vitro models of iENM toxicity assessment. In our evaluation, we found 6 clusters of factors deemed of relevance to studies of nanotoxicology of iENM: (i) using food-grade nanomaterials for toxicity testing; (ii) comprehensive PCM characterization of iENM in the dry state, (iii) standard NP dispersions and their characterization in cell culture media, (iv) determination of a realistic dose range, and its rationale; (v) in vitro dosimetry and in vitro – in vivo dose equivalencies; and (vi) investigation of dissolution kinetics and nanoparticle transformation. We further evaluated the most common test systems and endpoints reported. These factors, when not considered carefully, have the potential to influence and, at times, significantly alter the in vitro and in vivo testing results.

**ᅟ:** **SUMMARY Box 1 | Recommended considerations for toxicological investigation of ingested engineered nano/materials (iENM) and assessment of manuscripts during the peer-review process**

1. Test nano/materials should be food-grade (in vitro and in vivo) 2. They have been comprehensively characterized (in vitro and in vivo). 3. Nanoparticle dispersions should be prepared using standard and reproducible dispersion protocols (in vitro and in vivo). 4. Establish a realistic and physiologically-relevant dose range based on estimated daily intake, exposure variability, dose at the target site, and its estimated surface area (in vitro). Estimated daily intake values should be the basis for selection of relevant dose ranges in vivo studies. 5. Using appropriate co-culture or triculture models of gastrointestinal tract or microbiome that represent the exposure/target site (in vitro). 6. Confirmation of mature (intact/normal), immature (non-intact) or disease-state epithelium depending on the aim of the study (in vitro) 7. Consideration of the dynamics of the in vitro system (in vitro dosimetry). 8. Dissolution kinetics of test nanomaterial in relevant cell culture medium (in vitro). 9. Transformation of test nanomaterials through the gastrointestinal tract (if the target site is in the stomach, intestinal or colon segment) in the presence or absence of food matrix (in vitro).	

Although there is evidence that the authors of more recent literature are more mindful of such limitations, certain aspects of the studies, especially relevant test material, dose ranges, dosimetry and dissolution kinetics, continue to be overlooked.

For more relevant in vitro studies, it is important to use relevant food-grade nanomaterials that match the exposure scenario under investigation. More effort should be made to estimate nominal tissue doses using mathematical approaches that utilize estimated daily dietary intakes and physiologically relevant parameters such as tissue surface areas, residence time of iENM in various sections of the GI tract, and in vitro to in vivo dose equivalencies. Dissolution kinetics and biotransformation in relevant test medium are important to document for many iENM, especially ZnO, SiO_2_ and Fe_2_O_3_, including simulated or actual digestive fluids. Ingestion of ENM as a result of clearance mechanisms due to inhalation exposure should also be considered, especially in the context of occupational and consumer exposures. We encourage the use of the most physiologically relevant cell lines in recently developed tri-culture models representative of the complexity of intestinal mucosa and incorporating transformation and ENM-food interactions as they pass through the GI tract prior to doping the cells. Furthermore, future studies should also consider other subtler and less direct effects of iENM ingestion on the physiological functions of the GI tract, such as the effects of iENM ingestion on the antioxidant activity of foods, micronutrient absorption and their bioavailability, remodeling of the gut microbiome, and nanoparticle accumulation over long-term chronic exposures in other organs in humans, especially in liver and spleen. Chronic consumption of several iENM in foods should be incorporated in nutritional epidemiology and controlled human ingestion studies.

## Additional file

Additional file 1: **Table S1.** Important nanoparticle properties and common methods for characterization. (DOCX 14 kb)

## Abbreviations

Ag: Silver
ALT: Alanine aminotransferase
APT: Aspartate aminotransferase
CeO_2_: Cerium oxide
CePO_4_: Cerium phosphate
DLS: Dynamic light scattering
e-CFR: Electronic code of federal regulations
EFSA: European Food Safety Authority
ENM(s): Engineered nanomaterial(s)
EP: European Pharmacopeia
FDA: Food and Drug Administration
Fe_2_O_3_: Iron oxide
GI: Gastrointestinal
GRAS: Generally regarded as safe
HPMC: Hydropropylmethylcellulose
iENM(s): Ingested, food-grade engineered nanomaterial(s)
NF: National Formulary
NP(s): Nanoparticle(s)
NTA: Nanoparticle tracking analysis
OECD: Organization for Economic Co-operation and Development
PCM: Physicochemical and morphological
SiO_2_: Silicon dioxide
TEER: Trans-epithelial electrical resistance
TiO_2_: Titanium dioxide
USP: US Pharmacopeia
ZnO: Zinc oxide
