# Supplementary material for: Ingested engineered nanomaterials: state of science in nanotoxicity testing and future research needs
Source: Part Fibre Toxicol. 2018 Jul 3;15:29. doi: 10.1186/s12989-018-0265-1 (PMC6029122; doi:10.1186/s12989-018-0265-1)
Supplement: Supplementary file 1 — Table S1. Important nanoparticle properties and common methods for characterization. (DOCX 14 kb) [file 12989_2018_265_MOESM1_ESM.docx]

Table S1 - Important nanoparticle properties and common methods for characterization

| Physicochemical properties | Common characterization methods^a,b^ |
| --- | --- |
| Size (distribution) | TEM, AFM, DLS, NTA |
| Shape | TEM, AFM, UV-vis (for plasmonic nanoparticles) |
| Agglomeration or aggregation state | DLS, UV-vis (for plasmonic nanoparticles) |
| Crystal structure | XRD, ED |
| Surface chemistry/charge/area | AES, EELS, XPS, solid-state NMR, ζ-potential, BET |
| Stability over time/dissolution | DLS, UV-vis, ICP-AES, ICP-MS, colorimetric assays |
| Dosing metric |  |
| Uptake | ICP-AES, ICP-MS, TEM, fluorescence, flow cytometry, NAA |

^a^Abbreviations: TEM, transmission electron microscopy; AFM, atomic force microscopy; DLS, dynamic light scattering; NTA, nanoparticle-tracking analysis; UV-vis, UV-visible spectroscopy; XRD, X-ray diffraction; ED, electron diffraction; AES, Auger electron spectroscopy; EELS, electron energy loss spectroscopy; XPS, X-ray photoelectron spectroscopy; NMR, nuclear magnetic resonance; BET, nitrogen adsorption/desorption isotherm; ICP-AES, inductively-coupled plasma atomic emission spectroscopy; ICP-MS, inductively-coupled plasma mass spectrometry; NAA, neutron activation analysis.

^b^Not an exhaustive list of characterization approaches.
